# Stochastic synchronization of genetic oscillator networks

**DOI:** 10.1186/1752-0509-1-6

**Published:** 2007-01-09

**Authors:** Chunguang Li, Luonan Chen, Kazuyuki Aihara

**Affiliations:** 1 Centre for Nonlinear and Complex Systems, School of Electronic Engineering, University of Electronic Science and Technology of China, Chengdu 610054, P. R. China; 2 ERATO Aihara Complexity Modelling Project, Room M204, Komaba Open Laboratory, University of Tokyo, 4-6-1 Komaba, Meguro-ku, Tokyo 153-8505, Japan; 3 Institute of Industrial Science, University of Tokyo, Tokyo 153-8505, Japan; 4 Institute of Systems Biology, Shanghai University, P. R. China; 5 Department of Electrical Engineering and Electronics, Osaka Sanyo University, Osaka, Japan

## Abstract

**Background:**

The study of synchronization among genetic oscillators is essential for the understanding of the rhythmic phenomena of living organisms at both molecular and cellular levels. Genetic networks are intrinsically noisy due to natural random intra- and inter-cellular fluctuations. Therefore, it is important to study the effects of noise perturbation on the synchronous dynamics of genetic oscillators. From the synthetic biology viewpoint, it is also important to implement biological systems that minimizing the negative influence of the perturbations.

**Results:**

In this paper, based on systems biology approach, we provide a general theoretical result on the synchronization of genetic oscillators with stochastic perturbations. By exploiting the specific properties of many genetic oscillator models, we provide an easy-verified sufficient condition for the stochastic synchronization of coupled genetic oscillators, based on the Lur'e system approach in control theory. A design principle for minimizing the influence of noise is also presented. To demonstrate the effectiveness of our theoretical results, a population of coupled repressillators is adopted as a numerical example.

**Conclusion:**

In summary, we present an efficient theoretical method for analyzing the synchronization of genetic oscillator networks, which is helpful for understanding and testing the synchronization phenomena in biological organisms. Besides, the results are actually applicable to general oscillator networks.

## Background

Elucidating the collective dynamics of coupled genetic oscillators not only is important for the understanding of the rhythmic phenomena of living organisms, but also has many potential applications in bioengineering areas. So far, many researchers have studied the synchronization in genetic networks from the aspects of experiment, numerical simulation and theoretical analysis. For instance, in [1], the authors experimentally investigated the synchronization of cellular clock in the suprachiasmatic nucleus (SCN); in [2-4], the synchronization are studied in biological networks of identical genetic oscillators; and in [5-7], the synchronization for coupled nonidentical genetic oscillators is investigated. Gene regulation is an intrinsically noisy process, which is subject to intracellular and extracellular noise perturbations and environment fluctuations [8-12,14]. Such cellular noises will undoubtedly affect the dynamics of the networks both quantitatively and qualitatively. In [13], the authors numerically studied the cooperative behaviors of a multicell system with noise perturbations. But to our knowledge, the synchronization properties of stochastic genetic networks have not yet been theoretically studied.

This paper aims to provide a theoretical result for the synchronization of coupled genetic oscillators with noise perturbations, based on control theory approach. We first provide a general theoretical result for the stochastic synchronization of coupled oscillators. After that, by taking the specific structure of many model genetic oscillators into account, we present a sufficient condition for the stochastic synchronization in terms of linear matrix inequalities (LMIs) [15], which are very easy to be verified numerically. To our knowledge, the synchronization of complex oscillator networks with noise perturbations, even not in the biological context, has not yet been fully studied. Recently, it was found that many biological networks are complex networks with small-world and scale-free properties [16,17]. Our method is also applicable to genetic oscillator networks with complex topology, directed and weighted couplings. To demonstrate the effectiveness of the theoretical results, we present a simulation example of coupled repressilators. Throughout this paper, matrix *U *∈ *R*^*N *× *N *^is defined as an irreducible matrices with zero row sums, whose off-diagonal elements are all non-positive, and the other notations are defined in the Appendix A.

## Results

### Theoretical results

Since we know very little about how the cellular noises act on the genetic networks, a simple way to incorporate random effects is to assume that certain noises randomly perturb the genetic networks in an additive manner. We consider the following networks of *N *coupled genetic oscillators with random noise perturbations

dxi(t)dt=F(xi(t))+∑j=1NGijDxj(t)+vi(t)ni(t),  i=1,⋯,N
 MathType@MTEF@5@5@+=feaafiart1ev1aaatCvAUfKttLearuWrP9MDH5MBPbIqV92AaeXatLxBI9gBaebbnrfifHhDYfgasaacH8akY=wiFfYdH8Gipec8Eeeu0xXdbba9frFj0=OqFfea0dXdd9vqai=hGuQ8kuc9pgc9s8qqaq=dirpe0xb9q8qiLsFr0=vr0=vr0dc8meaabaqaciaacaGaaeqabaqabeGadaaakeaadaWcaaqaaiabdsgaKjabdIha4naaBaaaleaacqWGPbqAaeqaaOGaeiikaGIaemiDaqNaeiykaKcabaGaemizaqMaemiDaqhaaiabg2da9iabdAeagjabcIcaOiabdIha4naaBaaaleaacqWGPbqAaeqaaOGaeiikaGIaemiDaqNaeiykaKIaeiykaKIaey4kaSYaaabCaeaacqWGhbWrdaWgaaWcbaGaemyAaKMaemOAaOgabeaaaeaacqWGQbGAcqGH9aqpcqaIXaqmaeaacqWGobGta0GaeyyeIuoakiabdseaejabdIha4naaBaaaleaacqWGQbGAaeqaaOGaeiikaGIaemiDaqNaeiykaKIaey4kaSIaemODay3aaSbaaSqaaiabdMgaPbqabaGccqGGOaakcqWG0baDcqGGPaqkcqWGUbGBdaWgaaWcbaGaemyAaKgabeaakiabcIcaOiabdsha0jabcMcaPiabcYcaSiabbccaGiabbccaGiabdMgaPjabg2da9iabigdaXiabcYcaSiabl+UimjabcYcaSiabd6eaobaa@6B82@

where *F*(·) defines the dynamics of individual oscillators, *v*_*i*_(*t*) ∈ *R*^*n *× 1 ^is called the noise intensity vector, belongs to *L*_2 _[0, ∞). As we will see in the following analysis, the results hold no matter what *v*_*i*_(*t*) is and no matter where it is introduced, so we do not explicitly express the form of *v*_*i*_(*t*) here. *v*_*i*_(*t*) can also be a function of the variables (if so, some minor modifications are needed in the following). *n*_*i*_(*t*) is a scalar zero mean Gaussian white noise process. Recall that the time derivative of a Wiener process is a white noise process [19], hence we can define *dw*_*i*_(*t*) = *n*_*i*_(*t*)*dt*, where *w*_*i*_(*t*) is a scalar Wiener process. Thus, the above equation can be rewritten as the following stochastic differential equation form:

dxi(t)=[F(xi(t))+∑j=1NGijDxj(t)]dt+vi(t)dwi(t),  i=1,⋯,N.     (1)
 MathType@MTEF@5@5@+=feaafiart1ev1aaatCvAUfKttLearuWrP9MDH5MBPbIqV92AaeXatLxBI9gBaebbnrfifHhDYfgasaacH8akY=wiFfYdH8Gipec8Eeeu0xXdbba9frFj0=OqFfea0dXdd9vqai=hGuQ8kuc9pgc9s8qqaq=dirpe0xb9q8qiLsFr0=vr0=vr0dc8meaabaqaciaacaGaaeqabaqabeGadaaakeaacqWGKbazcqWG4baEdaWgaaWcbaGaemyAaKgabeaakiabcIcaOiabdsha0jabcMcaPiabg2da9maadmaabaGaemOrayKaeiikaGIaemiEaG3aaSbaaSqaaiabdMgaPbqabaGccqGGOaakcqWG0baDcqGGPaqkcqGGPaqkcqGHRaWkdaaeWbqaaiabdEeahnaaBaaaleaacqWGPbqAcqWGQbGAaeqaaOGaemiraqKaemiEaG3aaSbaaSqaaiabdQgaQbqabaaabaGaemOAaOMaeyypa0JaeGymaedabaGaemOta4eaniabggHiLdGccqGGOaakcqWG0baDcqGGPaqkaiaawUfacaGLDbaacqWGKbazcqWG0baDcqGHRaWkcqWG2bGDdaWgaaWcbaGaemyAaKgabeaakiabcIcaOiabdsha0jabcMcaPiabdsgaKjabdEha3naaBaaaleaacqWGPbqAaeqaaOGaeiikaGIaemiDaqNaeiykaKIaeiilaWIaeeiiaaIaeeiiaaIaemyAaKMaeyypa0JaeGymaeJaeiilaWIaeS47IWKaeiilaWIaemOta4KaeiOla4IaaCzcaiaaxMaadaqadaqaaiabigdaXaGaayjkaiaawMcaaaaa@7368@

The work can be easily extended to the case that *v*_*i*_(*t*) ∈ Rn×li
 MathType@MTEF@5@5@+=feaafiart1ev1aaatCvAUfKttLearuWrP9MDH5MBPbIqV92AaeXatLxBI9gBaebbnrfifHhDYfgasaacH8akY=wiFfYdH8Gipec8Eeeu0xXdbba9frFj0=OqFfea0dXdd9vqai=hGuQ8kuc9pgc9s8qqaq=dirpe0xb9q8qiLsFr0=vr0=vr0dc8meaabaqaciaacaGaaeqabaqabeGadaaakeaacqWGsbGudaahaaWcbeqaaiabd6gaUjabgEna0kabdYgaSnaaBaaameaacqWGPbqAaeqaaaaaaaa@346B@ and *n*_*i*_(*t*) = [*n*_*i*1_(*t*), ⋯, nili
 MathType@MTEF@5@5@+=feaafiart1ev1aaatCvAUfKttLearuWrP9MDH5MBPbIqV92AaeXatLxBI9gBaebbnrfifHhDYfgasaacH8akY=wiFfYdH8Gipec8Eeeu0xXdbba9frFj0=OqFfea0dXdd9vqai=hGuQ8kuc9pgc9s8qqaq=dirpe0xb9q8qiLsFr0=vr0=vr0dc8meaabaqaciaacaGaaeqabaqabeGadaaakeaacqWGUbGBdaWgaaWcbaGaemyAaKMaemiBaW2aaSbaaWqaaiabdMgaPbqabaaaleqaaaaa@328C@(*t*)]^*T *^be an *l*_*i*_-dimensional mutually independent zero mean Gaussian white noise process. *D *∈ *R*^*n *× *n *^defines the coupling between two genetic oscillators. *G *= (*G*_*ij*_)_*N *× *N *_is the coupling matrix of the network. If there is a link from oscillator *j *to oscillator *i *(*j *≠ *i*), then *G*_*ij *_equals to a positive constant denoting the coupling strength of this link; otherwise, *G*_*ij *_= 0; Gii=−∑j=1,j≠iNGij
 MathType@MTEF@5@5@+=feaafiart1ev1aaatCvAUfKttLearuWrP9MDH5MBPbIqV92AaeXatLxBI9gBaebbnrfifHhDYfgasaacH8akY=wiFfYdH8Gipec8Eeeu0xXdbba9frFj0=OqFfea0dXdd9vqai=hGuQ8kuc9pgc9s8qqaq=dirpe0xb9q8qiLsFr0=vr0=vr0dc8meaabaqaciaacaGaaeqabaqabeGadaaakeaacqWGhbWrdaWgaaWcbaGaemyAaKMaemyAaKgabeaakiabg2da9iabgkHiTmaaqadabaGaem4raC0aaSbaaSqaaiabdMgaPjabdQgaQbqabaaabaGaemOAaOMaeyypa0JaeGymaeJaeiilaWIaemOAaOMaeyiyIKRaemyAaKgabaGaemOta4eaniabggHiLdaaaa@426B@. Matrix *G *defines the coupling topology, direction, and the coupling strength of the network.

For network (1), a natural attempt is to study the mean-square asymptotic synchronization. But existing experimental results show that usually the genetic oscillators can not achieve mean-square synchronization (see, e.g. experimental results in [1] and Appendix B for a theoretical discussion). Analogue to the stochastic stability with disturbance attenuation (see, e.g. [20]), we give a less restrictive (but more realistic) definition of the stochastic synchronization as follows:

*Definition 1*: For a given scalar *γ *> 0, the network (1) is said to be stochastically synchronous (under the combination matrix *U*) with disturbance attenuation *γ *if the network without disturbance (*v*_*i *_= 0, ∀*i*) is asymptotically synchronous, and under the same initial conditions for all oscillators,

∑i<j(−Uij)‖xi(t)−xj(t)‖E22<γ∑i‖vi(t)‖22,     (2)
 MathType@MTEF@5@5@+=feaafiart1ev1aaatCvAUfKttLearuWrP9MDH5MBPbIqV92AaeXatLxBI9gBaebbnrfifHhDYfgasaacH8akY=wiFfYdH8Gipec8Eeeu0xXdbba9frFj0=OqFfea0dXdd9vqai=hGuQ8kuc9pgc9s8qqaq=dirpe0xb9q8qiLsFr0=vr0=vr0dc8meaabaqaciaacaGaaeqabaqabeGadaaakeaadaaeqbqaaiabcIcaOiabgkHiTiabdwfavnaaBaaaleaacqWGPbqAcqWGQbGAaeqaaOGaeiykaKYaauWaaeaacqWG4baEdaWgaaWcbaGaemyAaKgabeaakiabcIcaOiabdsha0jabcMcaPiabgkHiTiabdIha4naaBaaaleaacqWGQbGAaeqaaOGaeiikaGIaemiDaqNaeiykaKcacaGLjWUaayPcSdWaa0baaSqaaiabdweafnaaBaaameaacqaIYaGmaeqaaaWcbaGaeGOmaidaaOGaeyipaWdcciGae83SdC2aaabuaeaadaqbdaqaaiabdAha2naaBaaaleaacqWGPbqAaeqaaOGaeiikaGIaemiDaqNaeiykaKcacaGLjWUaayPcSdWaa0baaSqaaiabikdaYaqaaiabikdaYaaaaeaacqWGPbqAaeqaniabggHiLdaaleaacqWGPbqAcqGH8aapcqWGQbGAaeqaniabggHiLdGccqGGSaalcaWLjaGaaCzcamaabmaabaGaeGOmaidacaGLOaGaayzkaaaaaa@6350@

for all nonzero *v*_*i*_(*t*), where ‖⋅‖E22=E(∫0∞|⋅|2dt)
 MathType@MTEF@5@5@+=feaafiart1ev1aaatCvAUfKttLearuWrP9MDH5MBPbIqV92AaeXatLxBI9gBaebbnrfifHhDYfgasaacH8akY=wiFfYdH8Gipec8Eeeu0xXdbba9frFj0=OqFfea0dXdd9vqai=hGuQ8kuc9pgc9s8qqaq=dirpe0xb9q8qiLsFr0=vr0=vr0dc8meaabaqaciaacaGaaeqabaqabeGadaaakeaadaqbdaqaaiabgwSixdGaayzcSlaawQa7amaaDaaaleaacqWGfbqrdaWgaaadbaGaeGOmaidabeaaaSqaaiabikdaYaaakiabg2da9Gqabiab=veafnaabmaabaWaa8qmaeaadaabdaqaaiabgwSixdGaay5bSlaawIa7amaaCaaaleqabaGaeGOmaidaaOGaemizaqMaemiDaqhaleaacqaIWaamaeaacqGHEisPa0Gaey4kIipaaOGaayjkaiaawMcaaaaa@4714@. Here, by introducing the combination matrix *U*, we can flexibly select the form of the matrix to obtain different error combinations.

By using the techniques described in the Appendix C, we know that if there exist matrices *P *> 0, *T *∈ *R*^*n *× *n *^and *U*, and a scalar *ρ *> 0, such that the following conditions are satisfied,

S_2 _≡ 2(*y*_1 _- *y*_2_)^*T*^*P*[*F*(*y*_1_) - *F*(*y*_2_) - *T*(*y*_1 _- *y*_2_)] + ργ
 MathType@MTEF@5@5@+=feaafiart1ev1aaatCvAUfKttLearuWrP9MDH5MBPbIqV92AaeXatLxBI9gBaebbnrfifHhDYfgasaacH8akY=wiFfYdH8Gipec8Eeeu0xXdbba9frFj0=OqFfea0dXdd9vqai=hGuQ8kuc9pgc9s8qqaq=dirpe0xb9q8qiLsFr0=vr0=vr0dc8meaabaqaciaacaGaaeqabaqabeGadaaakeaadaWcaaqaaGGaciab=f8aYbqaaiab=n7aNbaaaaa@3025@(*y*_1 _- *y*_2_)^*T*^(*y*_1 _- *y*_2_) < 0, ∀*y*_1_, *y*_2 _∈ *R*^*n *^(*y*_1 _≠ *y*_2_),

(*U *⊗ *P*)(*G *⊗ *D *+ *I *⊗ *T*) + (*G *⊗ *D *+ *I *⊗ *T*)^*T*^(*U *⊗ *P*) ≤ 0,     (3)

*U *⊗ *P *≤ *ρI*,

then, the network (1) will achieve stochastic synchronization with disturbance attenuation *γ*.

The above condition (3) is a general result for the stochastic synchronization of coupled oscillators. But we do not have general efficient method for verifying the first inequality in (3) for arbitrary *F *due to its nonlinearity. Next we consider a special structure of genetic oscillators to obtain an easy-verified result.

Genetic oscillators are biochemically dynamical networks, which can usually be modelled as nonlinear dynamical systems. Mathematically many genetic oscillators can be expressed in the form of multiple additive terms, and the terms are monotonic functions of each variable, which particularly, are of linear, Michaelis-Menten and Hill forms. In our previous papers [7,23], we have taken such special structure properties of gene networks into account, and have shown that these genetic oscillators can be transformed into Lur'e form and can be further analyzed by using Lur'e system method in control theory [22]. In this paper, we also consider such special structure. To make our paper more understandable and self-contained, we will first introduce the approach briefly, and after that we will analyze the stochastic genetic oscillator networks theoretically. We consider the following general form of genetic oscillator:

y˙
 MathType@MTEF@5@5@+=feaafiart1ev1aaatCvAUfKttLearuWrP9MDH5MBPbIqV92AaeXatLxBI9gBaebbnrfifHhDYfgasaacH8akY=wiFfYdH8Gipec8Eeeu0xXdbba9frFj0=OqFfea0dXdd9vqai=hGuQ8kuc9pgc9s8qqaq=dirpe0xb9q8qiLsFr0=vr0=vr0dc8meaabaqaciaacaGaaeqabaqabeGadaaakeaacuWG5bqEgaGaaaaa@2E30@(*t*) = *Ay*(*t*) + *B*_1_*f*_1_(*y*(*t*)) + *B*_2_*f*_2_(*y*(*t*)),   (4)

where *y*(*t*) ∈ *R*^*n *^represents the concentrations of proteins, RNAs and chemical complexes, *A*, *B*_1 _and *B*_2 _are matrices in *R*^*n *× *n*^, *f*_1_(*y*(*t*)) = [*f*_11_(*y*_1_(*t*)), ⋯, *f*_1*n*_(*y*_*n*_(*t*))]^*T *^with *f*_1*j*_(*y_j_*(*t*)) as a monotonic increasing function of the form f1j(yj(t))=(yj(t)/β1j)H1j/[1+(yj(t)/β1j)H1j]
 MathType@MTEF@5@5@+=feaafiart1ev1aaatCvAUfKttLearuWrP9MDH5MBPbIqV92AaeXatLxBI9gBaebbnrfifHhDYfgasaacH8akY=wiFfYdH8Gipec8Eeeu0xXdbba9frFj0=OqFfea0dXdd9vqai=hGuQ8kuc9pgc9s8qqaq=dirpe0xb9q8qiLsFr0=vr0=vr0dc8meaabaqaciaacaGaaeqabaqabeGadaaakeaacqWGMbGzdaWgaaWcbaGaeGymaeJaemOAaOgabeaakiabcIcaOiabdMha5naaBaaaleaacqWGQbGAaeqaaOGaeiikaGIaemiDaqNaeiykaKIaeiykaKIaeyypa0JaeiikaGIaemyEaK3aaSbaaSqaaiabdQgaQbqabaGccqGGOaakcqWG0baDcqGGPaqkcqGGVaWliiGacqWFYoGydaWgaaWcbaGaeGymaeJaemOAaOgabeaakiabcMcaPmaaCaaaleqabaGaemisaG0aaSbaaWqaaiabigdaXiabdQgaQbqabaaaaOGaei4la8Iaei4waSLaeGymaeJaey4kaSIaeiikaGIaemyEaK3aaSbaaSqaaiabdQgaQbqabaGccqGGOaakcqWG0baDcqGGPaqkcqGGVaWlcqWFYoGydaWgaaWcbaGaeGymaeJaemOAaOgabeaakiabcMcaPmaaCaaaleqabaGaemisaG0aaSbaaWqaaiabigdaXiabdQgaQbqabaaaaOGaeiyxa0faaa@6015@, and *f*_2_(*y*(*t*)) = [*f*_21_(*y*_1_(*t*)), ⋯ , *f*_2*n*_(*y*_*n*_(*t*))]^*T *^with *f*_2*j*_(*y*_*j*_(*t*)) as a monotonic decreasing function of the form f2j(yj(t))=1/[1+(yj(t)/β2j)H2j]
 MathType@MTEF@5@5@+=feaafiart1ev1aaatCvAUfKttLearuWrP9MDH5MBPbIqV92AaeXatLxBI9gBaebbnrfifHhDYfgasaacH8akY=wiFfYdH8Gipec8Eeeu0xXdbba9frFj0=OqFfea0dXdd9vqai=hGuQ8kuc9pgc9s8qqaq=dirpe0xb9q8qiLsFr0=vr0=vr0dc8meaabaqaciaacaGaaeqabaqabeGadaaakeaacqWGMbGzdaWgaaWcbaGaeGOmaiJaemOAaOgabeaakiabcIcaOiabdMha5naaBaaaleaacqWGQbGAaeqaaOGaeiikaGIaemiDaqNaeiykaKIaeiykaKIaeyypa0JaeGymaeJaei4la8Iaei4waSLaeGymaeJaey4kaSIaeiikaGIaemyEaK3aaSbaaSqaaiabdQgaQbqabaGccqGGOaakcqWG0baDcqGGPaqkcqGGVaWliiGacqWFYoGydaWgaaWcbaGaeGOmaiJaemOAaOgabeaakiabcMcaPmaaCaaaleqabaGaemisaG0aaSbaaWqaaiabikdaYiabdQgaQbqabaaaaOGaeiyxa0faaa@5059@, where *H*_1*j *_and *H*_2*j *_are the Hill coefficients. Genetic oscillators of the form (4) is by no mean peculiar. Many well-known genetic system models can be represented in this form, such as the Goodwin model [24], the repressilator [25], the toggle switch [26], and the circadian oscillators [27]. Undoubtedly, this work can be easily generalized to the case of y˙(t)=Ay(t)+∑j=1lBjfj(y(t))
 MathType@MTEF@5@5@+=feaafiart1ev1aaatCvAUfKttLearuWrP9MDH5MBPbIqV92AaeXatLxBI9gBaebbnrfifHhDYfgasaacH8akY=wiFfYdH8Gipec8Eeeu0xXdbba9frFj0=OqFfea0dXdd9vqai=hGuQ8kuc9pgc9s8qqaq=dirpe0xb9q8qiLsFr0=vr0=vr0dc8meaabaqaciaacaGaaeqabaqabeGadaaakeaacuWG5bqEgaGaaiabcIcaOiabdsha0jabcMcaPiabg2da9iabdgeabjabdMha5jabcIcaOiabdsha0jabcMcaPiabgUcaRmaaqadabaGaemOqai0aaSbaaSqaaiabdQgaQbqabaGccqWGMbGzdaWgaaWcbaGaemOAaOgabeaaaeaacqWGQbGAcqGH9aqpcqaIXaqmaeaacqWGSbaBa0GaeyyeIuoakiabcIcaOiabdMha5jabcIcaOiabdsha0jabcMcaPiabcMcaPaaa@4B67@, where there are more than two nonlinear terms in each equation of the genetic oscillator. From the synthetic biology viewpoint, genetic oscillators with only linear, Michaelis-Menten and Hill terms can also be implemented experimentally.

To avoid confusion, we let the *j*th column of *B*_1,2 _be zeros if *f*_1*j*,2*j *_≡ 0. Since f2j(yj(t))=11+(yj(t)/β2j)H2j=1−(yj(t)/β2j)H2j1+(yj(t)/β2j)H2j≡1−gj(yj(t))
 MathType@MTEF@5@5@+=feaafiart1ev1aaatCvAUfKttLearuWrP9MDH5MBPbIqV92AaeXatLxBI9gBaebbnrfifHhDYfgasaacH8akY=wiFfYdH8Gipec8Eeeu0xXdbba9frFj0=OqFfea0dXdd9vqai=hGuQ8kuc9pgc9s8qqaq=dirpe0xb9q8qiLsFr0=vr0=vr0dc8meaabaqaciaacaGaaeqabaqabeGadaaakeaacqWGMbGzdaWgaaWcbaGaeGOmaiJaemOAaOgabeaakiabcIcaOiabdMha5naaBaaaleaacqWGQbGAaeqaaOGaeiikaGIaemiDaqNaeiykaKIaeiykaKIaeyypa0ZaaSaaaeaacqaIXaqmaeaacqaIXaqmcqGHRaWkcqGGOaakcqWG5bqEdaWgaaWcbaGaemOAaOgabeaakiabcIcaOiabdsha0jabcMcaPiabc+caVGGaciab=j7aInaaBaaaleaacqaIYaGmcqWGQbGAaeqaaOGaeiykaKYaaWbaaSqabeaacqWGibasdaWgaaadbaGaeGOmaiJaemOAaOgabeaaaaaaaOGaeyypa0JaeGymaeJaeyOeI0YaaSaaaeaacqGGOaakcqWG5bqEdaWgaaWcbaGaemOAaOgabeaakiabcIcaOiabdsha0jabcMcaPiabc+caViab=j7aInaaBaaaleaacqaIYaGmcqWGQbGAaeqaaOGaeiykaKYaaWbaaSqabeaacqWGibasdaWgaaadbaGaeGOmaiJaemOAaOgabeaaaaaakeaacqaIXaqmcqGHRaWkcqGGOaakcqWG5bqEdaWgaaWcbaGaemOAaOgabeaakiabcIcaOiabdsha0jabcMcaPiabc+caViab=j7aInaaBaaaleaacqaIYaGmcqWGQbGAaeqaaOGaeiykaKYaaWbaaSqabeaacqWGibasdaWgaaadbaGaeGOmaiJaemOAaOgabeaaaaaaaOGaeyyyIORaeGymaeJaeyOeI0Iaem4zaC2aaSbaaSqaaiabdQgaQbqabaGccqGGOaakcqWG5bqEdaWgaaWcbaGaemOAaOgabeaakiabcIcaOiabdsha0jabcMcaPiabcMcaPaaa@81A7@, and letting *f*(·) = *f*_1_(·), we can rewrite (4) as follows:

y˙
 MathType@MTEF@5@5@+=feaafiart1ev1aaatCvAUfKttLearuWrP9MDH5MBPbIqV92AaeXatLxBI9gBaebbnrfifHhDYfgasaacH8akY=wiFfYdH8Gipec8Eeeu0xXdbba9frFj0=OqFfea0dXdd9vqai=hGuQ8kuc9pgc9s8qqaq=dirpe0xb9q8qiLsFr0=vr0=vr0dc8meaabaqaciaacaGaaeqabaqabeGadaaakeaacuWG5bqEgaGaaaaa@2E30@(*t*) = *Ay*(*t*) + *B*_1_*f*(*y*(*t*)) - *B*_2_*g*(*y*(*t*)) + *B*_2_.    (5)

Obviously, *f*_*i *_and *g_i _*satisfy the sector conditions: 0≤fi(a)−fi(b)a−b<k1i,0≤gi(a)−gi(b)a−b≤k2i
 MathType@MTEF@5@5@+=feaafiart1ev1aaatCvAUfKttLearuWrP9MDH5MBPbIqV92AaeXatLxBI9gBaebbnrfifHhDYfgasaacH8akY=wiFfYdH8Gipec8Eeeu0xXdbba9frFj0=OqFfea0dXdd9vqai=hGuQ8kuc9pgc9s8qqaq=dirpe0xb9q8qiLsFr0=vr0=vr0dc8meaabaqaciaacaGaaeqabaqabeGadaaakeaacqaIWaamcqGHKjYOdaWcaaqaaiabdAgaMnaaBaaaleaacqWGPbqAaeqaaOGaeiikaGIaemyyaeMaeiykaKIaeyOeI0IaemOzay2aaSbaaSqaaiabdMgaPbqabaGccqGGOaakcqWGIbGycqGGPaqkaeaacqWGHbqycqGHsislcqWGIbGyaaGaeyipaWJaem4AaS2aaSbaaSqaaiabigdaXiabdMgaPbqabaGccqGGSaalcqaIWaamcqGHKjYOdaWcaaqaaiabdEgaNnaaBaaaleaacqWGPbqAaeqaaOGaeiikaGIaemyyaeMaeiykaKIaeyOeI0Iaem4zaC2aaSbaaSqaaiabdMgaPbqabaGccqGGOaakcqWGIbGycqGGPaqkaeaacqWGHbqycqGHsislcqWGIbGyaaGaeyizImQaem4AaS2aaSbaaSqaaiabikdaYiabdMgaPbqabaaaaa@5DDB@ or equivalently,

(*f*_*i*_(*a*) - *f*_*i*_(*b*)) [(*f*_*i*_(*a*) - *f*_*i*_(*b*)) - *k*_1*i*_(*a *- *b*)] ≤ 0,   (6)

(*g*_*i*_(*a*) - *g*_*i*_(*b*)) [*(g*_*i*_(*a*) - *g*_*i*_(*b*)) - *k*_2*i*_(*a *- *b*)] ≤ 0,

∀*a*, *b *∈ *R*(*a *≠ *b*);*i *= 1, ⋯, *n*,

Recall that a Lur'e system is a linear dynamic system, feedback interconnected to a static nonlinearity that satisfies a sector condition [22]. Hence, the genetic oscillator (5) can be seen as a Lur'e system, which can be investigated by using the fruitful Lur'e system approach in control theory.

By substituting the individual genetic oscillator dynamics (5) for *F *in the network (1), we obtain the following network of *N *coupled genetic oscillators:

dxi(t)=[Axi(t)+B1f(xi(t))−B2g(xi(t))+B2+∑j=1NGijDxj(t)]dt+vi(t)dwi(t),  i=1,⋯,N.     (7)
 MathType@MTEF@5@5@+=feaafiart1ev1aaatCvAUfKttLearuWrP9MDH5MBPbIqV92AaeXatLxBI9gBaebbnrfifHhDYfgasaacH8akY=wiFfYdH8Gipec8Eeeu0xXdbba9frFj0=OqFfea0dXdd9vqai=hGuQ8kuc9pgc9s8qqaq=dirpe0xb9q8qiLsFr0=vr0=vr0dc8meaabaqaciaacaGaaeqabaqabeGadaaakeaacqWGKbazcqWG4baEdaWgaaWcbaGaemyAaKgabeaakiabcIcaOiabdsha0jabcMcaPiabg2da9iabcUfaBjabdgeabjabdIha4naaBaaaleaacqWGPbqAaeqaaOGaeiikaGIaemiDaqNaeiykaKIaey4kaSIaemOqai0aaSbaaSqaaiabigdaXaqabaGccqWGMbGzcqGGOaakcqWG4baEdaWgaaWcbaGaemyAaKgabeaakiabcIcaOiabdsha0jabcMcaPiabcMcaPiabgkHiTiabdkeacnaaBaaaleaacqaIYaGmaeqaaOGaem4zaCMaeiikaGIaemiEaG3aaSbaaSqaaiabdMgaPbqabaGccqGGOaakcqWG0baDcqGGPaqkcqGGPaqkcqGHRaWkcqWGcbGqdaWgaaWcbaGaeGOmaidabeaakiabgUcaRmaaqadabaGaem4raC0aaSbaaSqaaiabdMgaPjabdQgaQbqabaaabaGaemOAaOMaeyypa0JaeGymaedabaGaemOta4eaniabggHiLdGccqWGebarcqWG4baEdaWgaaWcbaGaemOAaOgabeaakiabcIcaOiabdsha0jabcMcaPiabc2faDjabdsgaKjabdsha0jabgUcaRiabdAha2naaBaaaleaacqWGPbqAaeqaaOGaeiikaGIaemiDaqNaeiykaKIaemizaqMaem4DaC3aaSbaaSqaaiabdMgaPbqabaGccqGGOaakcqWG0baDcqGGPaqkcqGGSaalcqqGGaaicqqGGaaicqWGPbqAcqGH9aqpcqaIXaqmcqGGSaalcqWIVlctcqGGSaalcqWGobGtcqGGUaGlcaWLjaGaaCzcamaabmaabaGaeG4naCdacaGLOaGaayzkaaaaaa@8DBE@

For this network, we have the following result:

*Proposition 1*: If there are matrices *P *> 0, Λ_1 _= diag(λ_11_, ⋯,λ_1*n*_) > 0, Λ_2 _= diag(λ_21_, ⋯,λ_2*n*_) > 0, *Q *∈ *R*^*n *× *n*^, *U *as defined above, and a positive real constant *ρ *such that the following matrix inequalities hold

[(1,1)PB1+K1Λ1−PB2+K2Λ2B1TP+K1Λ1−2Λ10−B2TP+K2Λ20−2Λ2]<0,(UG⊗PD+U⊗Q)T+(UG⊗PD+U⊗Q)≤0,U⊗P≤ρI,     (8)
 MathType@MTEF@5@5@+=feaafiart1ev1aaatCvAUfKttLearuWrP9MDH5MBPbIqV92AaeXatLxBI9gBaebbnrfifHhDYfgasaacH8akY=wiFfYdH8Gipec8Eeeu0xXdbba9frFj0=OqFfea0dXdd9vqai=hGuQ8kuc9pgc9s8qqaq=dirpe0xb9q8qiLsFr0=vr0=vr0dc8meaabaqaciaacaGaaeqabaqabeGadaaakeaafaqaaeWabaaabaWaamWaaeaafaqabeWadaaabaGaeiikaGIaeGymaeJaeiilaWIaeGymaeJaeiykaKcabaGaemiuaaLaemOqai0aaSbaaSqaaiabigdaXaqabaGccqGHRaWkcqWGlbWsdaWgaaWcbaGaeGymaedabeaakiabfU5amnaaBaaaleaacqaIXaqmaeqaaaGcbaGaeyOeI0IaemiuaaLaemOqai0aaSbaaSqaaiabikdaYaqabaGccqGHRaWkcqWGlbWsdaWgaaWcbaGaeGOmaidabeaakiabfU5amnaaBaaaleaacqaIYaGmaeqaaaGcbaGaemOqai0aa0baaSqaaiabigdaXaqaaiabdsfaubaakiabdcfaqjabgUcaRiabdUealnaaBaaaleaacqaIXaqmaeqaaOGaeu4MdW0aaSbaaSqaaiabigdaXaqabaaakeaacqGHsislcqaIYaGmcqqHBoatdaWgaaWcbaGaeGymaedabeaaaOqaaiabicdaWaqaaiabgkHiTiabdkeacnaaDaaaleaacqaIYaGmaeaacqWGubavaaGccqWGqbaucqGHRaWkcqWGlbWsdaWgaaWcbaGaeGOmaidabeaakiabfU5amnaaBaaaleaacqaIYaGmaeqaaaGcbaGaeGimaadabaGaeyOeI0IaeGOmaiJaeu4MdW0aaSbaaSqaaiabikdaYaqabaaaaaGccaGLBbGaayzxaaGaeyipaWJaeGimaaJaeiilaWcabaGaeiikaGIaemyvauLaem4raCKaey4LIqSaemiuaaLaemiraqKaey4kaSIaemyvauLaey4LIqSaemyuaeLaeiykaKYaaWbaaSqabeaacqWGubavaaGccqGHRaWkcqGGOaakcqWGvbqvcqWGhbWrcqGHxkcXcqWGqbaucqWGebarcqGHRaWkcqWGvbqvcqGHxkcXcqWGrbqucqGGPaqkcqGHKjYOcqaIWaamcqGGSaalaeaacqWGvbqvcqGHxkcXcqWGqbaucqGHKjYOiiGacqWFbpGCcqWGjbqscqGGSaalaaGaaCzcaiaaxMaadaqadaqaaiabiIda4aGaayjkaiaawMcaaaaa@981F@

where (1,1) = *PA *+ *A*^*T*^*P *- *Q *- *Q*^*T *^+ ργ
 MathType@MTEF@5@5@+=feaafiart1ev1aaatCvAUfKttLearuWrP9MDH5MBPbIqV92AaeXatLxBI9gBaebbnrfifHhDYfgasaacH8akY=wiFfYdH8Gipec8Eeeu0xXdbba9frFj0=OqFfea0dXdd9vqai=hGuQ8kuc9pgc9s8qqaq=dirpe0xb9q8qiLsFr0=vr0=vr0dc8meaabaqaciaacaGaaeqabaqabeGadaaakeaadaWcaaqaaGGaciab=f8aYbqaaiab=n7aNbaaaaa@3025@*I*, *K*_1 _= diag(*k*_11_, ⋯, *k*_1*n*_), *K*_2 _= diag(*k*_21_, ⋯, *k*_2*n*_). Then the network (7) is stochastic synchronization with disturbance attenuation *γ*.

Proposition 1 can be proved by replacing *F *in S_2 _of (3) by the dynamics of (5), and using the sector conditions (6). The details are given in Appendix D. If we choose *U *beforehand, the matrix inequalities in (8) are all LMIs, which are very easy to be verified numerically [15]. For some special *G *and *D*, we can further simplify the verification process [21,7].

### An example

To demonstrate the effectiveness of our theoretical results, we consider a population of *N *coupled biological clocks, and the individual genetic oscillator is the repressilator [25]. The repressilator is a network of three genes, the products of which inhibit the transcription of each other in a cyclic way (10). Specifically, the gene *lacI *expresses protein LacI, which inhibits transcription of the gene *tetR*. The protein product TetR, inhibits transcription of the gene *cI*, the protein product CI of which in turn inhibits expression of *lacI*, thus forming a negative feedback cycle.

**Figure 1:**
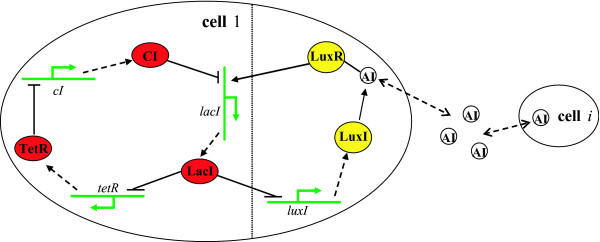
Schematic representation of the coupled repressilator network. In the left big circle, detailed regulation and coupling mechanism are presented. The repressilator module is located at the left of the vertical dotted line, and the coupling module appears at the right.

The quorum-sensing system is used for the coupling purpose, which was described in [5]. The system achieves cell-to-cell communication through a mechanism that makes use of two proteins, the first one of which (LuxI), under the control of the repressilator protein LacI, synthesizes a small molecule known as an autoinducer (AI), which can diffuse freely through the cell membrane. When a second protein (LuxR) binds to this molecule, the resulting complex activates the transcription *lacI*, as shown in Fig. 1. The noise perturbations in the model can arise both intracellularly, due to the intrinsically noisy property of the gene regulation process, and extracellularly, due to environment fluctuations.

To model the system, we use *a*_*i*_, *b*_*i*_, *c*_*i*_, and *A*_*i*_, *B*_*i*_, *C*_*i *_to represent the dimensionless concentrations of the genes *tetR, cI, lacI *and their product proteins TetR, CI, LacI, respectively. As in [5], assuming equal lifetimes of the TetR and Luxl proteins, their dynamics are identical, and hence we can use the same variable to describe both protein concentrations. The concentration of AI inside the *i*th cell is denoted by *S*_*i*_. Consequently, the mRNA and protein dynamics in the *i*th cell can be described by [5]:

daidt=−d1ai+αμ+Cim,dbidt=−d2bi+αμ+Aim,dcidt=−d3ci+αμ+Bim+kSiμs+Si,dAidt=−d4Ai+β1ai,dBidt=−d5Bi+β2bi,dCidt=−d6Ci+β3ci,dSidt=−ks0Si+ks1Ai+η(Se−Si),     (9)
 MathType@MTEF@5@5@+=feaafiart1ev1aaatCvAUfKttLearuWrP9MDH5MBPbIqV92AaeXatLxBI9gBaebbnrfifHhDYfgasaacH8akY=wiFfYdH8Gipec8Eeeu0xXdbba9frFj0=OqFfea0dXdd9vqai=hGuQ8kuc9pgc9s8qqaq=dirpe0xb9q8qiLsFr0=vr0=vr0dc8meaabaqaciaacaGaaeqabaqabeGadaaakeaafaqadeWbbaaaaeaadaWcaaqaaiabdsgaKjabdggaHnaaBaaaleaacqWGPbqAaeqaaaGcbaGaemizaqMaemiDaqhaaiabg2da9iabgkHiTiabdsgaKnaaBaaaleaacqaIXaqmaeqaaOGaemyyae2aaSbaaSqaaiabdMgaPbqabaGccqGHRaWkdaWcaaqaaGGaciab=f7aHbqaaiab=X7aTjabgUcaRiabdoeadnaaDaaaleaacqWGPbqAaeaacqWGTbqBaaaaaOGaeiilaWcabaWaaSaaaeaacqWGKbazcqWGIbGydaWgaaWcbaGaemyAaKgabeaaaOqaaiabdsgaKjabdsha0baacqGH9aqpcqGHsislcqWGKbazdaWgaaWcbaGaeGOmaidabeaakiabdkgaInaaBaaaleaacqWGPbqAaeqaaOGaey4kaSYaaSaaaeaacqWFXoqyaeaacqWF8oqBcqGHRaWkcqWGbbqqdaqhaaWcbaGaemyAaKgabaGaemyBa0gaaaaakiabcYcaSaqaamaalaaabaGaemizaqMaem4yam2aaSbaaSqaaiabdMgaPbqabaaakeaacqWGKbazcqWG0baDaaGaeyypa0JaeyOeI0Iaemizaq2aaSbaaSqaaiabiodaZaqabaGccqWGJbWydaWgaaWcbaGaemyAaKgabeaakiabgUcaRmaalaaabaGae8xSdegabaGae8hVd0Maey4kaSIaemOqai0aa0baaSqaaiabdMgaPbqaaiabd2gaTbaaaaGccqGHRaWkdaWcaaqaaiabdUgaRjabdofatnaaBaaaleaacqWGPbqAaeqaaaGcbaGae8hVd02aaSbaaSqaaiabdohaZbqabaGccqGHRaWkcqWGtbWudaWgaaWcbaGaemyAaKgabeaaaaGccqGGSaalaeaadaWcaaqaaiabdsgaKjabdgeabnaaBaaaleaacqWGPbqAaeqaaaGcbaGaemizaqMaemiDaqhaaiabg2da9iabgkHiTiabdsgaKnaaBaaaleaacqaI0aanaeqaaOGaemyqae0aaSbaaSqaaiabdMgaPbqabaGccqGHRaWkcqWFYoGydaWgaaWcbaGaeGymaedabeaakiabdggaHnaaBaaaleaacqWGPbqAaeqaaOGaeiilaWcabaWaaSaaaeaacqWGKbazcqWGcbGqdaWgaaWcbaGaemyAaKgabeaaaOqaaiabdsgaKjabdsha0baacqGH9aqpcqGHsislcqWGKbazdaWgaaWcbaGaeGynaudabeaakiabdkeacnaaBaaaleaacqWGPbqAaeqaaOGaey4kaSIae8NSdi2aaSbaaSqaaiabikdaYaqabaGccqWGIbGydaWgaaWcbaGaemyAaKgabeaakiabcYcaSaqaamaalaaabaGaemizaqMaem4qam0aaSbaaSqaaiabdMgaPbqabaaakeaacqWGKbazcqWG0baDaaGaeyypa0JaeyOeI0Iaemizaq2aaSbaaSqaaiabiAda2aqabaGccqWGdbWqdaWgaaWcbaGaemyAaKgabeaakiabgUcaRiab=j7aInaaBaaaleaacqaIZaWmaeqaaOGaem4yam2aaSbaaSqaaiabdMgaPbqabaGccqGGSaalaeaadaWcaaqaaiabdsgaKjabdofatnaaBaaaleaacqWGPbqAaeqaaaGcbaGaemizaqMaemiDaqhaaiabg2da9iabgkHiTiabdUgaRnaaBaaaleaacqWGZbWCcqaIWaamaeqaaOGaem4uam1aaSbaaSqaaiabdMgaPbqabaGccqGHRaWkcqWGRbWAdaWgaaWcbaGaem4CamNaeGymaedabeaakiabdgeabnaaBaaaleaacqWGPbqAaeqaaOGaey4kaSIae83TdGMaeiikaGIaem4uam1aaSbaaSqaaiabdwgaLbqabaGccqGHsislcqWGtbWudaWgaaWcbaGaemyAaKgabeaakiabcMcaPiabcYcaSaaacaWLjaGaaCzcamaabmaabaGaeGyoaKdacaGLOaGaayzkaaaaaa@E7A4@

where *m *is the Hill coefficient, *S*_*e *_denotes the extracellular AI concentration, and the meaning of the other parameters are standard in genetic network models. We assume that the release of the AI is fast with respect to the timescale of the oscillators and becomes approximately homogeneous to establish an average AI level outside the cells. In the quasi-steady-state approximation, the extracellular AI concentration can be approximated by [5]

Se=Q0S¯=Q0N∑j=1NSj,
 MathType@MTEF@5@5@+=feaafiart1ev1aaatCvAUfKttLearuWrP9MDH5MBPbIqV92AaeXatLxBI9gBaebbnrfifHhDYfgasaacH8akY=wiFfYdH8Gipec8Eeeu0xXdbba9frFj0=OqFfea0dXdd9vqai=hGuQ8kuc9pgc9s8qqaq=dirpe0xb9q8qiLsFr0=vr0=vr0dc8meaabaqaciaacaGaaeqabaqabeGadaaakeaacqWGtbWudaWgaaWcbaGaemyzaugabeaakiabg2da9iabdgfarnaaBaaaleaacqaIWaamaeqaaOGafm4uamLbaebacqGH9aqpdaWcaaqaaiabdgfarnaaBaaaleaacqaIWaamaeqaaaGcbaGaemOta4eaamaaqahabaGaem4uam1aaSbaaSqaaiabdQgaQbqabaaabaGaemOAaOMaeyypa0JaeGymaedabaGaemOta4eaniabggHiLdGccqGGSaalaaa@42DB@

where 0 <*Q*_0 _< 1 is a constant. Thus the dynamics of *S_i _*can be rewritten as

dSidt=−[ks0+(1−Q0)η]Si+ks1Ai+ηQ0N∑j=1N(Sj−Si)     (10)
 MathType@MTEF@5@5@+=feaafiart1ev1aaatCvAUfKttLearuWrP9MDH5MBPbIqV92AaeXatLxBI9gBaebbnrfifHhDYfgasaacH8akY=wiFfYdH8Gipec8Eeeu0xXdbba9frFj0=OqFfea0dXdd9vqai=hGuQ8kuc9pgc9s8qqaq=dirpe0xb9q8qiLsFr0=vr0=vr0dc8meaabaqaciaacaGaaeqabaqabeGadaaakeaadaWcaaqaaiabdsgaKjabdofatnaaBaaaleaacqWGPbqAaeqaaaGcbaGaemizaqMaemiDaqhaaiabg2da9iabgkHiTiabcUfaBjabdUgaRnaaBaaaleaacqWGZbWCcqaIWaamaeqaaOGaey4kaSIaeiikaGIaeGymaeJaeyOeI0Iaemyuae1aaSbaaSqaaiabicdaWaqabaGccqGGPaqkiiGacqWF3oaAcqGGDbqxcqWGtbWudaWgaaWcbaGaemyAaKgabeaakiabgUcaRiabdUgaRnaaBaaaleaacqWGZbWCcqaIXaqmaeqaaOGaemyqae0aaSbaaSqaaiabdMgaPbqabaGccqGHRaWkdaWcaaqaaiab=D7aOjabdgfarnaaBaaaleaacqaIWaamaeqaaaGcbaGaemOta4eaamaaqahabaGaeiikaGIaem4uam1aaSbaaSqaaiabdQgaQbqabaGccqGHsislcqWGtbWudaWgaaWcbaGaemyAaKgabeaakiabcMcaPaWcbaGaemOAaOMaeyypa0JaeGymaedabaGaemOta4eaniabggHiLdGccaWLjaGaaCzcamaabmaabaGaeGymaeJaeGimaadacaGLOaGaayzkaaaaaa@6836@

Clearly, the individual model in (9) is of the form (5), in which *f *= [0, 0, 0, 0, 0, 0, *S*_*i*_/(*μ_s _*+ *S*_*i*_)]^*T*^, *B*_1 _is a 7 × 7 matrix with all zero entries except for B_1_(3, 7) = *k*, *g *= [0, 0, 0, Aim
 MathType@MTEF@5@5@+=feaafiart1ev1aaatCvAUfKttLearuWrP9MDH5MBPbIqV92AaeXatLxBI9gBaebbnrfifHhDYfgasaacH8akY=wiFfYdH8Gipec8Eeeu0xXdbba9frFj0=OqFfea0dXdd9vqai=hGuQ8kuc9pgc9s8qqaq=dirpe0xb9q8qiLsFr0=vr0=vr0dc8meaabaqaciaacaGaaeqabaqabeGadaaakeaacqWGbbqqdaqhaaWcbaGaemyAaKgabaGaemyBa0gaaaaa@30A2@/(*μ *+ Aim
 MathType@MTEF@5@5@+=feaafiart1ev1aaatCvAUfKttLearuWrP9MDH5MBPbIqV92AaeXatLxBI9gBaebbnrfifHhDYfgasaacH8akY=wiFfYdH8Gipec8Eeeu0xXdbba9frFj0=OqFfea0dXdd9vqai=hGuQ8kuc9pgc9s8qqaq=dirpe0xb9q8qiLsFr0=vr0=vr0dc8meaabaqaciaacaGaaeqabaqabeGadaaakeaacqWGbbqqdaqhaaWcbaGaemyAaKgabaGaemyBa0gaaaaa@30A2@), Bim
 MathType@MTEF@5@5@+=feaafiart1ev1aaatCvAUfKttLearuWrP9MDH5MBPbIqV92AaeXatLxBI9gBaebbnrfifHhDYfgasaacH8akY=wiFfYdH8Gipec8Eeeu0xXdbba9frFj0=OqFfea0dXdd9vqai=hGuQ8kuc9pgc9s8qqaq=dirpe0xb9q8qiLsFr0=vr0=vr0dc8meaabaqaciaacaGaaeqabaqabeGadaaakeaacqWGcbGqdaqhaaWcbaGaemyAaKgabaGaemyBa0gaaaaa@30A4@/(*μ *+Bim
 MathType@MTEF@5@5@+=feaafiart1ev1aaatCvAUfKttLearuWrP9MDH5MBPbIqV92AaeXatLxBI9gBaebbnrfifHhDYfgasaacH8akY=wiFfYdH8Gipec8Eeeu0xXdbba9frFj0=OqFfea0dXdd9vqai=hGuQ8kuc9pgc9s8qqaq=dirpe0xb9q8qiLsFr0=vr0=vr0dc8meaabaqaciaacaGaaeqabaqabeGadaaakeaacqWGcbGqdaqhaaWcbaGaemyAaKgabaGaemyBa0gaaaaa@30A4@), Cim
 MathType@MTEF@5@5@+=feaafiart1ev1aaatCvAUfKttLearuWrP9MDH5MBPbIqV92AaeXatLxBI9gBaebbnrfifHhDYfgasaacH8akY=wiFfYdH8Gipec8Eeeu0xXdbba9frFj0=OqFfea0dXdd9vqai=hGuQ8kuc9pgc9s8qqaq=dirpe0xb9q8qiLsFr0=vr0=vr0dc8meaabaqaciaacaGaaeqabaqabeGadaaakeaacqWGdbWqdaqhaaWcbaGaemyAaKgabaGaemyBa0gaaaaa@30A6@/(*μ *+ Cim
 MathType@MTEF@5@5@+=feaafiart1ev1aaatCvAUfKttLearuWrP9MDH5MBPbIqV92AaeXatLxBI9gBaebbnrfifHhDYfgasaacH8akY=wiFfYdH8Gipec8Eeeu0xXdbba9frFj0=OqFfea0dXdd9vqai=hGuQ8kuc9pgc9s8qqaq=dirpe0xb9q8qiLsFr0=vr0=vr0dc8meaabaqaciaacaGaaeqabaqabeGadaaakeaacqWGdbWqdaqhaaWcbaGaemyAaKgabaGaemyBa0gaaaaa@30A6@), 0]^*T*^, *B*_2 _is a 7 × 7 matrix with all zero entries except for *B*_2_(1,6) = *B*_2_(2, 4) = *B*_2_(3, 5) = *α*/*μ*, and all the other terms are in linear form. Obviously, the coupling term can also be written into the form defined previously.

**Figure 2:**
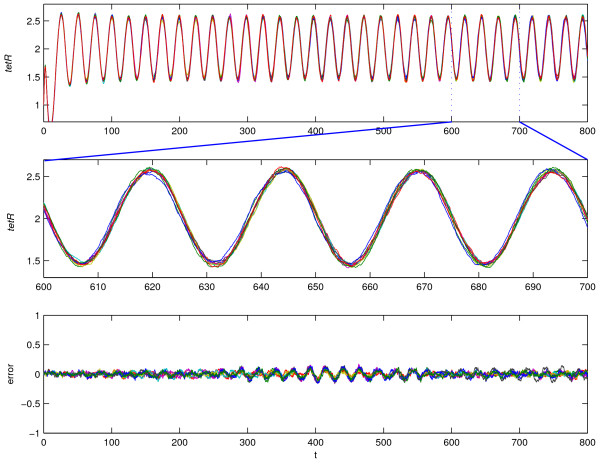
Simulation results of the coupled repressilators with the same initial values. (a) The evolution dynamics of the mRNA concentrations of *tetR *(*a*_*i*_) of all the genetic oscillators. (b) Zooming in the range *t *∈ [600, 700] of (a). (c) The evolution of the synchronization error of *a*_*i *_- *a*_1 _for *i *= 2, ⋯ ,10.

The purpose of this example is to demonstrate the effectiveness and correctness of the theoretical result, instead of mimicking the real biological clock system. We consider a small size of network with *N *= 10 coupled oscillators. The parameters are set as *m *= 4, *α *= 1.8, *d*_1 _= *d*_2 _= *d*_3 _= 0.4, *μ *= 1.3, *k *= 5, *μ*_*s *_= 5, *d*_4 _= *d*_5 _= *d*_6 _= 0.5, *β*_1 _= *β*_2 _= *β*_3 _= 0.2, *k*_*s*0 _= 0.016, *k*_*s*1 _= 0.018, *Q*_0 _= 0.8 and *η *= 0.4. Since Proposition 1 holds no matter what *v*_*i*_(*t*) is and no matter where it is introduced, and the verification of Proposition 1 is independent of noise intensity *v*_*i*_, for simplicity, we set *v*_*i *_= 0.015 as a scalar for all *i*, and the noise term *v*_*i*_*n*_*i*_(*t*) is added to the first equation in (9), where *n*_*i*_(*t*) is a scalar Gaussian white noise process. According to Proposition 1 (by letting *U *= -*G*, and using MATLAB LMI Toolbox), we know that the above all-to-all coupled network can achieve stochastic synchronization with disturbance attenuation *γ *= 6. Although *γ *is a large value, it is easy to show from (2) that the time average of **E**(∑_*i*_∑_*j*_|*x*_*i*_(*t*) - *x*_*j*_(*t*)|^2^) is still rather small because vi2
 MathType@MTEF@5@5@+=feaafiart1ev1aaatCvAUfKttLearuWrP9MDH5MBPbIqV92AaeXatLxBI9gBaebbnrfifHhDYfgasaacH8akY=wiFfYdH8Gipec8Eeeu0xXdbba9frFj0=OqFfea0dXdd9vqai=hGuQ8kuc9pgc9s8qqaq=dirpe0xb9q8qiLsFr0=vr0=vr0dc8meaabaqaciaacaGaaeqabaqabeGadaaakeaacqWG2bGDdaqhaaWcbaGaemyAaKgabaGaeGOmaidaaaaa@309B@ is very small. We omit the computational details here. In Fig. 2(a) &2(b), when starting from the same initial values, we plot the time evolution of the mRNA concentrations of *tetR *(*a*_*i*_) of all the oscillators, which behaviors are similar to the experimental results (see, e.g. [1]). Fig. 2(c) shows the synchronization error of *a*_*i *_- *a*_1 _for *i *= 2, ⋯, 10.

In Definition 1, it requires that all the genetic oscillators have the same initial conditions, so that *V*(*x*(0)) = 0. If the genetic oscillators have different initial conditions, *V*(*x*(0)) ≠ 0, and thus (12) in the Appendix C is replaced by

J(t)≤E{∫0t[αLV(x(s))+∑i<j(−Uij)(xi(s)−xj(s))T(xi(s)−xj(s))−γ∑iviT(s)vi(s)]ds}+E(V(x(0)).
 MathType@MTEF@5@5@+=feaafiart1ev1aaatCvAUfKttLearuWrP9MDH5MBPbIqV92AaeXatLxBI9gBaebbnrfifHhDYfgasaacH8akY=wiFfYdH8Gipec8Eeeu0xXdbba9frFj0=OqFfea0dXdd9vqai=hGuQ8kuc9pgc9s8qqaq=dirpe0xb9q8qiLsFr0=vr0=vr0dc8meaabaqaciaacaGaaeqabaqabeGadaaakeaacqWGkbGscqGGOaakcqWG0baDcqGGPaqkcqGHKjYOieqacqWFfbqrcqGG7bWEdaWdXaqaaiabcUfaBHGaciab+f7aHjabdYeamjabdAfawbWcbaGaeGimaadabaGaemiDaqhaniabgUIiYdGccqGGOaakcqWG4baEcqGGOaakcqWGZbWCcqGGPaqkcqGGPaqkcqGHRaWkdaaeqaqaaiabcIcaOiabgkHiTiabdwfavnaaBaaaleaacqWGPbqAcqWGQbGAaeqaaOGaeiykaKIaeiikaGIaemiEaG3aaSbaaSqaaiabdMgaPbqabaGccqGGOaakcqWGZbWCcqGGPaqkcqGHsislcqWG4baEdaWgaaWcbaGaemOAaOgabeaakiabcIcaOiabdohaZjabcMcaPiabcMcaPmaaCaaaleqabaGaemivaqfaaOGaeiikaGIaemiEaG3aaSbaaSqaaiabdMgaPbqabaGccqGGOaakcqWGZbWCcqGGPaqkcqGHsislcqWG4baEdaWgaaWcbaGaemOAaOgabeaakiabcIcaOiabdohaZjabcMcaPiabcMcaPiabgkHiTiab+n7aNnaaqababaGaemODay3aa0baaSqaaiabdMgaPbqaaiabdsfaubaakiabcIcaOiabdohaZjabcMcaPiabdAha2naaBaaaleaacqWGPbqAaeqaaOGaeiikaGIaem4CamNaeiykaKIaeiyxa0LaemizaqMaem4CamhaleaacqWGPbqAaeqaniabggHiLdaaleaacqWGPbqAcqGH8aapcqWGQbGAaeqaniabggHiLdGccqGG9bqFcqGHRaWkcqWFfbqrcqGGOaakcqWGwbGvcqGGOaakcqWG4baEcqGGOaakcqaIWaamcqGGPaqkcqGGPaqkcqGGUaGlaaa@956D@

**Figure 3:**
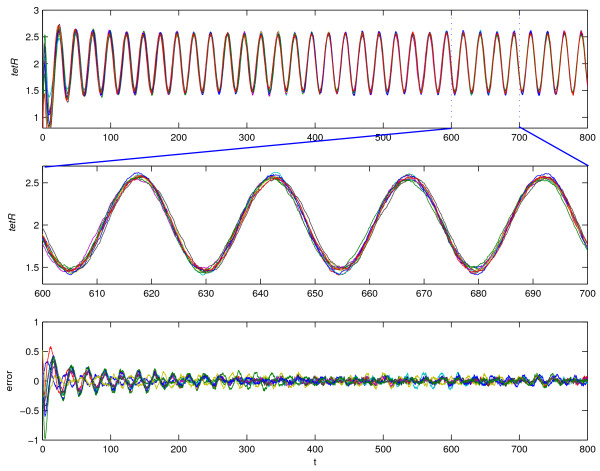
Coupled genetic oscillators with different initial conditions: (a) The evolution dynamics of the mRNA concentrations of *tetR *(*a*_*i*_) of all the genetic oscillators. (b) Zooming in the range *t *∈ [600, 700] of (a). (c) The evolution of the synchronization error of *a*_*i *_- *a*_1 _for *i *= 2, ⋯ ,10.

Since in genetic networks, the variables usually represent the concentrations of mRNAs, proteins and chemical complexes, which are of (not so large) limited values, and so is *V*(*x*(0)). For a long time scale, the last term of the above inequality is usually much smaller than the absolute value of the first term in the right-hand side, and thus the last term can be ignored roughly. In Fig. 3, we show the same computations as those in Fig. 2 except that the oscillators are with different initial values (randomly in the range (0, 1)). After a period of evolution, the network behaviors are similar to those in Fig. 2, which verifies our above argument. In other words, rigorously, according to Definition 1, we need that all the oscillators have the same initial conditions, but practically, for oscillators with different initial conditions, we can obtain almost the same results.

**Figure 4:**
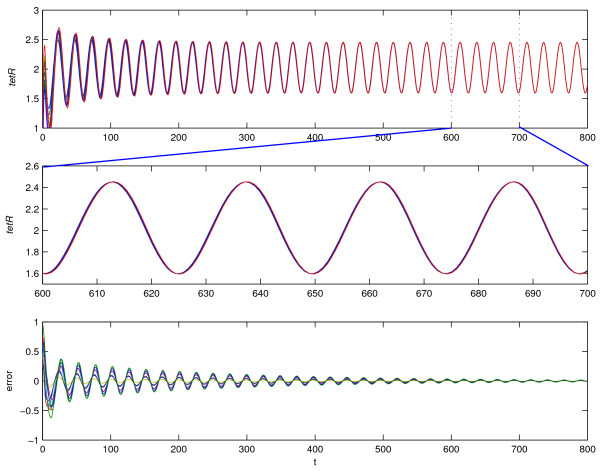
Coupled genetic oscillators without noise perturbation: (a) The evolution dynamics of the mRNA concentrations of *tetR *(*a*_*i*_) of all the genetic oscillators. (b) Zooming in the range *t *∈ [600, 700] of (a). (c) The evolution of the synchronization error of *a*_*i *_- *a*_1 _for *i *= 2, ⋯ ,10.

For the purpose of comparison, in Fig. 4 we show the simulation results of a network without noise perturbations. As we can conclude from Figs. 2, 3, 4, the networks with noise perturbation, though can't achieve perfect synchronization, can indeed achieve synchronization with small error fluctuation, and the network behaviors are similar to those of networks without noise perturbations.

### Synthesis

In addition to providing a sufficient condition for the stochastic synchronization, Proposition 1 can also be used for designing genetic oscillator networks, which is a byproduct of the main results. From the synthetic biology viewpoint, to minimize the influence of the noises (on the synchronization), we can design genetic oscillator networks according to the following rule:

min *γ*, such that the LMIs (8) hold,    (11)

which is obviously from the above theoretical result. This is similar to the *H*_∞ _synthesis problem in control theory.

## Discussion and conclusion

In this paper, we presented a general theoretical method for analyzing the stochastic synchronization of coupled genetic oscillators based on systems biology approach. By taking the specific structure of genetic systems into account, a sufficient condition for the stochastic synchronization was derived based on LMI formalism, which can be easily verified numerically. Although the method and results are presented for genetic oscillator networks, it is also applicable to other dynamical systems. In coupled genetic oscillator networks, since there is a maximal activity of fully active promoters, it is more realistic to consider a Michaelis-Menten form of the coupling terms. As argued in [7], our theoretical method is also applicable to this case. To make the theoretical method more understandable and to avoid unnecessarily complicated notation, we discussed only on some simplified forms of the genetic oscillators, but more general cases regarding this topic can be studied in a similar way. For example: (I) The genetic oscillator model (5) can be generalized to more general case such that *f*_*i*_, *g*_*i*_, the component of *f*(*y*(*t*)), *g*(*y*(*t*)), are functions of *y*(*t*), not only of *y*_*i*_(*t*), and *f *and *g *can also be of non-Hill form, provided that 0≤fi(a)−fi(b)c1iT(a−b)≤k1i,0≤gi(a)−gi(b)c2iT(a−b)≤k2i
 MathType@MTEF@5@5@+=feaafiart1ev1aaatCvAUfKttLearuWrP9MDH5MBPbIqV92AaeXatLxBI9gBaebbnrfifHhDYfgasaacH8akY=wiFfYdH8Gipec8Eeeu0xXdbba9frFj0=OqFfea0dXdd9vqai=hGuQ8kuc9pgc9s8qqaq=dirpe0xb9q8qiLsFr0=vr0=vr0dc8meaabaqaciaacaGaaeqabaqabeGadaaakeaacqaIWaamcqGHKjYOdaWcaaqaaiabdAgaMnaaBaaaleaacqWGPbqAaeqaaOGaeiikaGIaemyyaeMaeiykaKIaeyOeI0IaemOzay2aaSbaaSqaaiabdMgaPbqabaGccqGGOaakcqWGIbGycqGGPaqkaeaacqWGJbWydaqhaaWcbaGaeGymaeJaemyAaKgabaGaemivaqfaaOGaeiikaGIaemyyaeMaeyOeI0IaemOyaiMaeiykaKcaaiabgsMiJkabdUgaRnaaBaaaleaacqaIXaqmcqWGPbqAaeqaaOGaeiilaWIaeGimaaJaeyizIm6aaSaaaeaacqWGNbWzdaWgaaWcbaGaemyAaKgabeaakiabcIcaOiabdggaHjabcMcaPiabgkHiTiabdEgaNnaaBaaaleaacqWGPbqAaeqaaOGaeiikaGIaemOyaiMaeiykaKcabaGaem4yam2aa0baaSqaaiabikdaYiabdMgaPbqaaiabdsfaubaakiabcIcaOiabdggaHjabgkHiTiabdkgaIjabcMcaPaaacqGHKjYOcqWGRbWAdaWgaaWcbaGaeGOmaiJaemyAaKgabeaaaaa@6BF6@, ∀*a*, *b *∈ *R*^*n *^(*a *≠ *b*), *i *= 1, ⋯ , *n*, where *c*_1*i*_, *c*_2*i *_∈ *R*^*n *^are real vectors. (II) Biologically, the genetic oscillators are usually nonidentical. We can consider genetic networks with both parametric mismatches and stochastic perturbations in similar ways as those presented in this paper and [7]. (III) There are significant time delays in the gene regulation, due to the slow processes of transcription, translation and translocation. Our result can be easily extended to the case that there are delays both in the coupling and the individual genetic oscillators.

As we know, noises can play both beneficial and harmful roles (for synchronization) in biological systems. For the latter case, the noise is a kind of perturbation, and it is interesting to study the robustness of the synchronization with respect to noise. In this paper, we addressed this question. For the former case, in [13,14], the authors studied the mechanisms of noise-induced synchronization.

## Methods

To simulate the stocahstic differential equaiton x˙
 MathType@MTEF@5@5@+=feaafiart1ev1aaatCvAUfKttLearuWrP9MDH5MBPbIqV92AaeXatLxBI9gBaebbnrfifHhDYfgasaacH8akY=wiFfYdH8Gipec8Eeeu0xXdbba9frFj0=OqFfea0dXdd9vqai=hGuQ8kuc9pgc9s8qqaq=dirpe0xb9q8qiLsFr0=vr0=vr0dc8meaabaqaciaacaGaaeqabaqabeGadaaakeaacuWG4baEgaGaaaaa@2E2E@(*t*) = *f*(*x*) + *g*(*x*)*ξ*(*t*), the well-known Euler-Maruyama scheme is most frequently used, which is also used in this paper. In this scheme, the numerical trajectory is generated by *x*_*n*+1 _= *x*_*n *_+ *hf*(*x*_*n*_) + h
 MathType@MTEF@5@5@+=feaafiart1ev1aaatCvAUfKttLearuWrP9MDH5MBPbIqV92AaeXatLxBI9gBaebbnrfifHhDYfgasaacH8akY=wiFfYdH8Gipec8Eeeu0xXdbba9frFj0=OqFfea0dXdd9vqai=hGuQ8kuc9pgc9s8qqaq=dirpe0xb9q8qiLsFr0=vr0=vr0dc8meaabaqaciaacaGaaeqabaqabeGadaaakeaadaGcaaqaaiabdIgaObWcbeaaaaa@2E20@*g*(*x*_*n*_)*η*_*n*_, where *h *is the time step and *η*_*n *_is a discrete time Gaussian white noise with <*η*_*n *_>= 0 and <*η*_*n*_*η*_*m *_> = *δ*_*nm*_. For more details, see e.g. [18].

## Appendices

### A. Notations

Throughout this paper, *A*^*T *^denotes the transpose of a square matrix *A*. The notation *M *> (<) 0 is used to define a real symmetric positive definite (negative definite) matrix. *R*^*m *^denotes the *m*-dimensional Euclidean space; and *R*^*n *× *m *^denotes the set of all *n *× *m *real matrices. In this paper, if not explicitly stated, matrices are assumed to have compatible dimensions. **E**(·) denotes the expectation operator; *L*_2_[0, ∞) is the space of square-integrable vector functions over [0, ∞); |·| stands for the Euclidean vector norm, and ||·||_2 _stands for the usual *L*_2_[0, ∞) norm. The Kronecker product *A *⊗ *B *of an *n *× *m *matrix *A *and a *p *× *q *matrix *B *is the *np *× *mq *matrix defined as

A⊗B=[A11B⋯A1mB⋮⋱⋮An1B⋯AnmB].
 MathType@MTEF@5@5@+=feaafiart1ev1aaatCvAUfKttLearuWrP9MDH5MBPbIqV92AaeXatLxBI9gBaebbnrfifHhDYfgasaacH8akY=wiFfYdH8Gipec8Eeeu0xXdbba9frFj0=OqFfea0dXdd9vqai=hGuQ8kuc9pgc9s8qqaq=dirpe0xb9q8qiLsFr0=vr0=vr0dc8meaabaqaciaacaGaaeqabaqabeGadaaakeaacqWGbbqqcqGHxkcXcqWGcbGqcqGH9aqpdaWadaqaauaabeqadmaaaeaacqWGbbqqdaWgaaWcbaGaeGymaeJaeGymaedabeaakiabdkeacbqaaiabl+UimbqaaiabdgeabnaaBaaaleaacqaIXaqmcqWGTbqBaeqaaOGaemOqaieabaGaeSO7I0eabaGaeSy8I8eabaGaeSO7I0eabaGaemyqae0aaSbaaSqaaiabd6gaUjabigdaXaqabaGccqWGcbGqaeaacqWIVlctaeaacqWGbbqqdaWgaaWcbaGaemOBa4MaemyBa0gabeaakiabdkeacbaaaiaawUfacaGLDbaacqGGUaGlaaa@50F3@

For a general stochastic systems

*dx *= *f*(*t*, *x*(*t*))*dt *+ *g*(*x*(*t*))*dw*(*t*),

the diffusion operator *L *acting on *Y*(*t*, *x*(*t*)) is defined by

*LY*(*t*, *x*(*t*)) = *Y*_*t*_(*t*, *x*(*t*)) + *Y*_*x*_(*t*, *x*(*t*))*f*(*t*, *x*(*t*)) + 12
 MathType@MTEF@5@5@+=feaafiart1ev1aaatCvAUfKttLearuWrP9MDH5MBPbIqV92AaeXatLxBI9gBaebbnrfifHhDYfgasaacH8akY=wiFfYdH8Gipec8Eeeu0xXdbba9frFj0=OqFfea0dXdd9vqai=hGuQ8kuc9pgc9s8qqaq=dirpe0xb9q8qiLsFr0=vr0=vr0dc8meaabaqaciaacaGaaeqabaqabeGadaaakeaadaWcaaqaaiabigdaXaqaaiabikdaYaaaaaa@2E9E@trace[*g*(*x*(*t*))*g*^*T*^(*x*(*t*))*Y*_*xx*_(*t*, *x*(*t*))].

### B. Mean-square synchronization

For network (1), a natural attempt is to study the mean-square asymptotic synchronization. Analogue to the definition of mean-square stability [19], we can define the mean-square synchronization as follows:

*Definition A1*: The network (1) is said to be mean-square synchronous if for every *ε *> 0, there is a *δ*(*ε*) > 0, such that E|xi(t)−xj(t)|t>02
 MathType@MTEF@5@5@+=feaafiart1ev1aaatCvAUfKttLearuWrP9MDH5MBPbIqV92AaeXatLxBI9gBaebbnrfifHhDYfgasaacH8akY=wiFfYdH8Gipec8Eeeu0xXdbba9frFj0=OqFfea0dXdd9vqai=hGuQ8kuc9pgc9s8qqaq=dirpe0xb9q8qiLsFr0=vr0=vr0dc8meaabaqaciaacaGaaeqabaqabeGadaaakeaaieqacqWFfbqrdaabdaqaaiabdIha4naaBaaaleaacqWGPbqAaeqaaOGaeiikaGIaemiDaqNaeiykaKIaeyOeI0IaemiEaG3aaSbaaSqaaiabdQgaQbqabaGccqGGOaakcqWG0baDcqGGPaqkaiaawEa7caGLiWoadaqhaaWcbaGaemiDaqNaeyOpa4JaeGimaadabaGaeGOmaidaaaaa@42B6@ <*ε *for |*x*_*i*_(0) - *x*_*j*_(0)| <*δ*(*ε*), ∀*i*, *j*. If in addition, lim_*t*→∞ _**E**|*x_i_*(*t*) - *x_j_*(*t*)|^2 ^= 0 for all initial conditions, the network is said to be mean square asymptotically synchronous.

In analyzing the synchronization of the network (1), we use the Lyapunov function *V*(*x*(*t*)) = *x*^*T*^(*t*)(*U *⊗ *P*)*x*(*t*) [21], where ⊗ is the Kronecker product, and x(t)=[x1T(t),⋯,xnT(t)]T∈RNn×1
 MathType@MTEF@5@5@+=feaafiart1ev1aaatCvAUfKttLearuWrP9MDH5MBPbIqV92AaeXatLxBI9gBaebbnrfifHhDYfgasaacH8akY=wiFfYdH8Gipec8Eeeu0xXdbba9frFj0=OqFfea0dXdd9vqai=hGuQ8kuc9pgc9s8qqaq=dirpe0xb9q8qiLsFr0=vr0=vr0dc8meaabaqaciaacaGaaeqabaqabeGadaaakeaacqWG4baEcqGGOaakcqWG0baDcqGGPaqkcqGH9aqpcqGGBbWwcqWG4baEdaqhaaWcbaGaeGymaedabaGaemivaqfaaOGaeiikaGIaemiDaqNaeiykaKIaeiilaWIaeS47IWKaeiilaWIaemiEaG3aa0baaSqaaiabd6gaUbqaaiabdsfaubaakiabcIcaOiabdsha0jabcMcaPiabc2faDnaaCaaaleqabaGaemivaqfaaOGaeyicI4SaemOuai1aaWbaaSqabeaacqWGobGtcqWGUbGBcqGHxdaTcqaIXaqmaaaaaa@50B0@. According to [21], this Lyapunov function is equivalent to *V*(*x*(*t*)) = ∑_*i *<*j*_(-*U*_*ij*_)(*x*_*i*_(*t*) - *x*_*j*_(*t*))^*T*^*P*(*x*_*i*_(*t*) - *x*_*j*_(*t*)).

By It*ô*'s formula [19], we obtain the following stochastic differential along (1)

*dV*(*x*(*t*)) = *LV*(*x*(*t*))*dt *+ 2*x*^*T*^(*t*)(*U *⊗ *P*)*v*(*t*)*dw*(*t*)

where *v*(*t*) = diag(*v*_1_, ⋯, *v*_*N*_) ∈ *R*^*Nn *× *N*^, *L *is the diffusion operator, and

*LV*(*x*(*t*)) = 2∑_*i *<*j*_(-*U*_*ij*_)(*x*_*i *_- *x*_*j*_)^*T*^*P*[*F*(*x*_*i*_) - *F*(*x*_*j*_) - *T*(*x*_*i *_- *x*_*j*_)]

+ 2*x*^*T*^(*t*)(*U *⊗ *P*)(*G *⊗ *D *+ *I *⊗ *T*)*x*(*t*)

+ trace(*v*(*t*)*v*^*T*^(*t*)(*U *⊗ *P*))

We discuss two special cases of the stochastic terms:

1. The genetic oscillators are perturbed by the same noise, which can occur in the situation that genetic oscillators communicate via a common environment. In this case, *v*_*i*_*d*_*wi *_are the same for all *i*. We let [v=[viT,⋯,vNT]T
 MathType@MTEF@5@5@+=feaafiart1ev1aaatCvAUfKttLearuWrP9MDH5MBPbIqV92AaeXatLxBI9gBaebbnrfifHhDYfgasaacH8akY=wiFfYdH8Gipec8Eeeu0xXdbba9frFj0=OqFfea0dXdd9vqai=hGuQ8kuc9pgc9s8qqaq=dirpe0xb9q8qiLsFr0=vr0=vr0dc8meaabaqaciaacaGaaeqabaqabeGadaaakeaacqWG2bGDcqGH9aqpcqGGBbWwcqWG2bGDdaqhaaWcbaGaemyAaKgabaGaemivaqfaaOGaeiilaWIaeS47IWKaeiilaWIaemODay3aa0baaSqaaiabd6eaobqaaiabdsfaubaakiabc2faDnaaCaaaleqabaGaemivaqfaaaaa@3EED@] and *dw *= *dw*_*i *_Since *U *is a matrix with zero row sums and *v*_*i *_is the same for all *i*, it is easy to show that the last term of *LV *is zero. Thus if the following conditions hold, we will have **E**[*dV*(*x*(*t*))] = **E**[*LV*(*x*(*t*))*dt*] < 0.

(*y*_1 _- *y*_2_)^*T*^*P*[*F*(*y*_1_) - *F*(*y*_2_) - *T*(*y*_1 _- *y*_2_)] < 0,

∀ *y*_1_, *y*_2 _∈ *R*^*n *^(*y*_1 _≠ *y*_2_)

(*U *⊗ *P*)(*G *⊗ *D *+ *I *⊗ *T*) + (*G *⊗ *D *+ *I *⊗ *T*)^*T*^(*U *⊗ *P*) ≤ 0.

Hence, if there are matrices *P *> 0, *T *∈ *R*^*n *× *n *^and *U*, such that the above conditions hold, the network (1) will achieve mean-square asymptotically synchronization. In this case, roughly speaking, the noise will not affect the synchronous state (since they are common for all oscillators), but it will affect the individual oscillator dynamics.

2. The noise intensity matrix *v*_*i *_is a function of ∑_*j *_*G*_*ij*_*D*_*xj*_, which means that if there is no coupling from oscillator *j *to *i*, then *j *does not have contribution to the perturbation of oscillator *i*. We further assume that *v*_*i *_can be estimated by

viTvi≤[∑jGijDxj(t)]THi[∑jGijDxj(t)],  Hi≥0.
 MathType@MTEF@5@5@+=feaafiart1ev1aaatCvAUfKttLearuWrP9MDH5MBPbIqV92AaeXatLxBI9gBaebbnrfifHhDYfgasaacH8akY=wiFfYdH8Gipec8Eeeu0xXdbba9frFj0=OqFfea0dXdd9vqai=hGuQ8kuc9pgc9s8qqaq=dirpe0xb9q8qiLsFr0=vr0=vr0dc8meaabaqaciaacaGaaeqabaqabeGadaaakeaacqWG2bGDdaqhaaWcbaGaemyAaKgabaGaemivaqfaaOGaemODay3aaSbaaSqaaiabdMgaPbqabaGccqGHKjYOdaWadaqaamaaqafabaGaem4raC0aaSbaaSqaaiabdMgaPjabdQgaQbqabaGccqWGebarcqWG4baEdaWgaaWcbaGaemOAaOgabeaakiabcIcaOiabdsha0jabcMcaPaWcbaGaemOAaOgabeqdcqGHris5aaGccaGLBbGaayzxaaWaaWbaaSqabeaacqWGubavaaGccqWGibasdaWgaaWcbaGaemyAaKgabeaakmaadmaabaWaaabuaeaacqWGhbWrdaWgaaWcbaGaemyAaKMaemOAaOgabeaakiabdseaejabdIha4naaBaaaleaacqWGQbGAaeqaaOGaeiikaGIaemiDaqNaeiykaKcaleaacqWGQbGAaeqaniabggHiLdaakiaawUfacaGLDbaacqGGSaalcqqGGaaicqqGGaaicqWGibasdaWgaaWcbaGaemyAaKgabeaakiabgwMiZkabicdaWiabc6caUaaa@63E3@

Defining *v *= diag(*v*_1_, ⋯, *v*_*N*_) ∈ *R*^*Nn *× *N*^, *w*(*t*) = [*w*_1_(*t*), ⋯, *w*_*N*_(*t*)]^*T*^, and *H *= diag(*H*_1_, ⋯, *H*_*N*_), and assuming *U *⊗ *P *≤ *ρI*, we have

trace(*v*(*t*)*v*^*T*^(*t*)(*U *⊗ *P*)) ≤ λ_*max*_(*U *⊗ *P*)trace(*v*(*t*)*v*^*T*^(*t*))

≤ *ρ*∑_*i*_viT
 MathType@MTEF@5@5@+=feaafiart1ev1aaatCvAUfKttLearuWrP9MDH5MBPbIqV92AaeXatLxBI9gBaebbnrfifHhDYfgasaacH8akY=wiFfYdH8Gipec8Eeeu0xXdbba9frFj0=OqFfea0dXdd9vqai=hGuQ8kuc9pgc9s8qqaq=dirpe0xb9q8qiLsFr0=vr0=vr0dc8meaabaqaciaacaGaaeqabaqabeGadaaakeaacqWG2bGDdaqhaaWcbaGaemyAaKgabaGaemivaqfaaaaa@30DA@(*t*)*v*_*i*_(*t*)

≤ *ρx*^*T*^(*t*) (*G *⊗ *D*)^*T *^*H*(*G *⊗ *D*)*x*(*t*).

So, the conditions for the mean-square asymptotically synchronization of the network (1) in this case are

(*y*_1 _- *y*_2_)^*T*^*P*[*F*(*y*_1_) - *F*(*y*_2_) - *T*(*y*_1 _- *y*_2_)] < 0,

∀*y*_1_, *y*_2 _∈ *R*^*n *^(*y*_1 _≠ *y*_2_)

(*U *⊗ *P*)(*G *⊗ *D *+ *I *⊗ *T*) + (*G *⊗ *D *+ *I *⊗ *T*)^*T*^(*U *⊗ *P*) + *ρ*(*G *⊗ *D*)^*T*^*H*(*G *⊗ *D*) ≤ 0,

*U *⊗ *P *≤ *ρI*.

If we consider genetic oscillators of the form of (5), the conditions for the mean-square asymptotically synchronization can be analyzed by the same method as that in the following Appendix D.

From Definition Al, we know that the definition of the mean-square asymptotically synchronization is rather restrictive, which requires that lim_*t*→∞ _**E**|*x*_*i*_(*t*) - *x*_*j*_(*t*)|^2 ^= 0, ∀*i*, *j*. If it is neither of the above two cases, that is neither *V*_*i*_*d*_*wi *_are the same for all *i*, nor *v*_*i *_(∀*i*) reduce to zero when *x*_1 _= ⋯ = *x*_*n*_, the network is hardly to achieve mean-square asymptotically synchronization. Experimental results also show that usually the genetic oscillators can not achieve mean-square synchronization (see for example [1]). So, we argue that the study of mean-square synchronization is unrealistic (and therefore meaningless) in genetic networks.

In Ref. [28], the authors studied the mean-square asymptotic synchronization of two master-slave coupled Chua's circuits. They assume that the noise intensity depends on the difference of the states of the two systems, which is also somewhat unrealistic.

### C. Analysis of the general synchronization condition

To obtain the general synchronization condition (3) of the network (1), we also use the Lyapunov function *V*(*x*(*t*)) = *x*^*T*^(*t*)(*U *⊗ *P*)*x*(*t*). By It*ô*s formula [19], we obtain the stochastic differential *dV*(*x*(*t*))*= LV*(*x*(*t*))*dt *+ 2*x*^*T*^(*t*)(*U *⊗ *P*)*v*(*t*)*dw*(*t*). According to Definition 1, we assume that the oscillators have the same initial conditions, thus we can derive **E**(*V*(*x*(*t*)) = (∫0tLV(x(s))ds)
 MathType@MTEF@5@5@+=feaafiart1ev1aaatCvAUfKttLearuWrP9MDH5MBPbIqV92AaeXatLxBI9gBaebbnrfifHhDYfgasaacH8akY=wiFfYdH8Gipec8Eeeu0xXdbba9frFj0=OqFfea0dXdd9vqai=hGuQ8kuc9pgc9s8qqaq=dirpe0xb9q8qiLsFr0=vr0=vr0dc8meaabaqaciaacaGaaeqabaqabeGadaaakeaadaqadaqaamaapedabaGaemitaWKaemOvayfaleaacqaIWaamaeaacqWG0baDa0Gaey4kIipakiabcIcaOiabdIha4jabcIcaOiabdohaZjabcMcaPiabcMcaPiabdsgaKjabdohaZbGaayjkaiaawMcaaaaa@3E29@. For *γ *> 0, we define

J(t)=E{∫0t[∑i<j(−Uij)(xi(s)−xj(s))T(xi(s)−xj(s))−γ∑iviT(s)vi(s)]ds}     (12)
 MathType@MTEF@5@5@+=feaafiart1ev1aaatCvAUfKttLearuWrP9MDH5MBPbIqV92AaeXatLxBI9gBaebbnrfifHhDYfgasaacH8akY=wiFfYdH8Gipec8Eeeu0xXdbba9frFj0=OqFfea0dXdd9vqai=hGuQ8kuc9pgc9s8qqaq=dirpe0xb9q8qiLsFr0=vr0=vr0dc8meaabaqaciaacaGaaeqabaqabeGadaaakeaacqWGkbGscqGGOaakcqWG0baDcqGGPaqkcqGH9aqpieqacqWFfbqrcqGG7bWEdaWdXaqaaiabcUfaBnaaqafabaGaeiikaGIaeyOeI0Iaemyvau1aaSbaaSqaaiabdMgaPjabdQgaQbqabaGccqGGPaqkcqGGOaakcqWG4baEdaWgaaWcbaGaemyAaKgabeaakiabcIcaOiabdohaZjabcMcaPaWcbaGaemyAaKMaeyipaWJaemOAaOgabeqdcqGHris5aaWcbaGaeGimaadabaGaemiDaqhaniabgUIiYdGccqGHsislcqWG4baEdaWgaaWcbaGaemOAaOgabeaakiabcIcaOiabdohaZjabcMcaPiabcMcaPmaaCaaaleqabaGaemivaqfaaOGaeiikaGIaemiEaG3aaSbaaSqaaiabdMgaPbqabaGccqGGOaakcqWGZbWCcqGGPaqkcqGHsislcqWG4baEdaWgaaWcbaGaemOAaOgabeaakiabcIcaOiabdohaZjabcMcaPiabcMcaPiabgkHiTGGaciab+n7aNnaaqafabaGaemODay3aa0baaSqaaiabdMgaPbqaaiabdsfaubaakiabcIcaOiabdohaZjabcMcaPiabdAha2naaBaaaleaacqWGPbqAaeqaaOGaeiikaGIaem4CamNaeiykaKIaeiyxa0LaemizaqMaem4CamNaeiyFa0haleaacqWGPbqAaeqaniabggHiLdGccaWLjaGaaCzcamaabmaabaGaeGymaeJaeGOmaidacaGLOaGaayzkaaaaaa@8423@

Then, it is easy to show that for *α *> 0,

J(t)≤E{∫0t[αLV(x(s))+∑i<j(−Uij)(xi(s)−xj(s))T     ⋅(xi(s)−xj(s))−γ∑iviT(s)vi(s)]ds}≡E{∫0tS1(s)ds}.     (13)
 MathType@MTEF@5@5@+=feaafiart1ev1aaatCvAUfKttLearuWrP9MDH5MBPbIqV92AaeXatLxBI9gBaebbnrfifHhDYfgasaacH8akY=wiFfYdH8Gipec8Eeeu0xXdbba9frFj0=OqFfea0dXdd9vqai=hGuQ8kuc9pgc9s8qqaq=dirpe0xb9q8qiLsFr0=vr0=vr0dc8meaabaqaciaacaGaaeqabaqabeGadaaakeGaeiatciaUciasdeaLluaabmqadeaaaeaacqWGkbGscqGGOaakcqWG0baDcqGGPaqkcqGHKjYOieqacqWFfbqrcqGG7bWEdaWdXaqaaiabcUfaBHGaciab+f7aHjabdYeamjabdAfawjabcIcaOiabdIha4jabcIcaOiabdohaZjabcMcaPiabgUcaRmaaqababaGaeiikaGIaeyOeI0Iaemyvau1aaSbaaSqaaiabdMgaPjabdQgaQbqabaGccqGGPaqkcqGGOaakcqWG4baEdaWgaaWcbaGaemyAaKgabeaakiabcIcaOiabdohaZjabcMcaPiabgkHiTiabdIha4naaBaaaleaacqWGQbGAaeqaaOGaeiikaGIaem4CamNaeiykaKcaleaacqWGPbqAcqGH8aapcqWGQbGAaeqaniabggHiLdaaleaacqaIWaamaeaacqWG0baDa0Gaey4kIipakiabcMcaPmaaCaaaleqabaGaemivaqfaaaGcbiGaaeiab0yccaWLjaGaaCzcaiabgwSixlabcIcaOiabdIha4naaBaaaleaacqWGPbqAaeqaaOGaeiikaGIaem4CamNaeiykaKIaeyOeI0IaemiEaG3aaSbaaSqaaiabdQgaQbqabaGccqGGOaakcqWGZbWCcqGGPaqkcqGGPaqkcqGHsislcqGFZoWzdaaeqaqaaiabdAha2naaDaaaleaacqWGPbqAaeaacqWGubavaaaabaGaemyAaKgabeqdcqGHris5aOGaeiikaGIaem4CamNaeiykaKIaemODay3aaSbaaSqaaiabdMgaPbqabaGccqGGOaakcqWGZbWCcqGGPaqkcqGGDbqxcqWGKbazcqWGZbWCcqGG9bqFaeaacqGHHjIUcqWFfbqrcqGG7bWEdaWdXaqaaiabdofatnaaBaaaleaacqaIXaqmaeqaaaqaaiabicdaWaqaaiabdsha0bqdcqGHRiI8aOGaeiikaGIaem4CamNaeiykaKIaemizaqMaem4CamNaeiyFa0NaeiOla4caaiaaxMaacaWLjaWaaeWaaeaacqaIXaqmcqaIZaWmaiaawIcacaGLPaaaaaa@A8AB@

Assuming *U *⊗ *P *≤ *ρI*, and letting *α *= *γ/ρ*, we have

S1≡γρ{2∑i<j(−Uij)(xi(t)−xj(t))T⋅P[F(xi(t))−F(xj(t))−T(xi(t)−xj(t))]+2xT(t)(U⊗P)(G⊗D+I⊗T)x(t)+trace(v(t)vT(t)(U⊗P))+ργ∑i<j(−Uij)(xi(t)−xj(t))T(xi(t)−xj(t))−ρ∑iviT(t)vi(t)}≤γρ{∑i<j(−Uij)[2(xi(t)−xj(t))T⋅P(F(xi)−F(xj)−T(xi−xj))+ργ(xi(t)−xj(t))T(xi(t)−xj(t))]+2xT(t)(U⊗P)(G⊗D+I⊗T)x(t)}.
 MathType@MTEF@5@5@+=feaafiart1ev1aaatCvAUfKttLearuWrP9MDH5MBPbIqV92AaeXatLxBI9gBaebbnrfifHhDYfgasaacH8akY=wiFfYdH8Gipec8Eeeu0xXdbba9frFj0=OqFfea0dXdd9vqai=hGuQ8kuc9pgc9s8qqaq=dirpe0xb9q8qiLsFr0=vr0=vr0dc8meaabaqaciaacaGaaeqabaqabeGadaaakeaafaqadeGcdaaaaaqaaiabdofatnaaBaaaleaacqaIXaqmaeqaaaGcbiqaaSWbcqGHHjIUaeaadaWcaaqaaGGaciab=n7aNbqaaiab=f8aYbaacqGG7bWEcqaIYaGmdaaeqaqaaiabcIcaOiabgkHiTiabdwfavnaaBaaaleaacqWGPbqAcqWGQbGAaeqaaOGaeiykaKIaeiikaGIaemiEaG3aaSbaaSqaaiabdMgaPbqabaGccqGGOaakcqWG0baDcqGGPaqkcqGHsislcqWG4baEdaWgaaWcbaGaemOAaOgabeaakiabcIcaOiabdsha0jabcMcaPiabcMcaPmaaCaaaleqabaGaemivaqfaaaqaaiabdMgaPjabgYda8iabdQgaQbqab0GaeyyeIuoaaOqaaaqaaaqaaiabgwSixlabdcfaqjabcUfaBjabdAeagjabcIcaOiabdIha4naaBaaaleaacqWGPbqAaeqaaOGaeiikaGIaemiDaqNaeiykaKIaeiykaKIaeyOeI0IaemOrayKaeiikaGIaemiEaG3aaSbaaSqaaiabdQgaQbqabaGccqGGOaakcqWG0baDcqGGPaqkcqGGPaqkcqGHsislcqWGubavcqGGOaakcqWG4baEdaWgaaWcbaGaemyAaKgabeaakiabcIcaOiabdsha0jabcMcaPiabgkHiTiabdIha4naaBaaaleaacqWGQbGAaeqaaOGaeiikaGIaemiDaqNaeiykaKIaeiykaKIaeiyxa0fabaaabaaabaGaey4kaSIaeGOmaiJaemiEaG3aaWbaaSqabeaacqWGubavaaGccqGGOaakcqWG0baDcqGGPaqkcqGGOaakcqWGvbqvcqGHxkcXcqWGqbaucqGGPaqkcqGGOaakcqWGhbWrcqGHxkcXcqWGebarcqGHRaWkcqWGjbqscqGHxkcXcqWGubavcqGGPaqkcqWG4baEcqGGOaakcqWG0baDcqGGPaqkaeaaaeaaaeaacqGHRaWkcqqG0baDcqqGYbGCcqqGHbqycqqGJbWycqqGLbqzcqGGOaakcqWG2bGDcqGGOaakcqWG0baDcqGGPaqkcqWG2bGDdaahaaWcbeqaaiabdsfaubaakiabcIcaOiabdsha0jabcMcaPiabcIcaOiabdwfavjabgEPielabdcfaqjabcMcaPiabcMcaPaqaaaqaaaqaaiabgUcaRmaalaaabaGae8xWdihabaGae83SdCgaamaaqababaGaeiikaGIaeyOeI0Iaemyvau1aaSbaaSqaaiabdMgaPjabdQgaQbqabaGccqGGPaqkcqGGOaakcqWG4baEdaWgaaWcbaGaemyAaKgabeaakiabcIcaOiabdsha0jabcMcaPiabgkHiTiabdIha4naaBaaaleaacqWGQbGAaeqaaOGaeiikaGIaemiDaqNaeiykaKIaeiykaKcaleaacqWGPbqAcqGH8aapcqWGQbGAaeqaniabggHiLdGcdaahaaWcbeqaaiabdsfaubaakiabcIcaOiabdIha4naaBaaaleaacqWGPbqAaeqaaOGaeiikaGIaemiDaqNaeiykaKIaeyOeI0IaemiEaG3aaSbaaSqaaiabdQgaQbqabaGccqGGOaakcqWG0baDcqGGPaqkcqGGPaqkaeaaaeaaaeaacqGHsislcqWFbpGCdaaeqaqaaiabdAha2naaDaaaleaacqWGPbqAaeaacqWGubavaaaabaGaemyAaKgabeqdcqGHris5aOGaeiikaGIaemiDaqNaeiykaKIaemODay3aaSbaaSqaaiabdMgaPbqabaGccqGGOaakcqWG0baDcqGGPaqkcqGG9bqFaeaaaeaacqGHKjYOaeaadaWcaaqaaiab=n7aNbqaaiab=f8aYbaacqGG7bWEdaaeqaqaaiabcIcaOiabgkHiTiabdwfavnaaBaaaleaacqWGPbqAcqWGQbGAaeqaaOGaeiykaKIaei4waSLaeGOmaiJaeiikaGIaemiEaG3aaSbaaSqaaiabdMgaPbqabaGccqGGOaakcqWG0baDcqGGPaqkcqGHsislcqWG4baEdaWgaaWcbaGaemOAaOgabeaakiabcIcaOiabdsha0jabcMcaPiabcMcaPmaaCaaaleqabaGaemivaqfaaaqaaiabdMgaPjabgYda8iabdQgaQbqab0GaeyyeIuoaaOqaaaqaaaqaaiabgwSixlabdcfaqjabcIcaOiabdAeagjabcIcaOiabdIha4naaBaaaleaacqWGPbqAaeqaaOGaeiykaKIaeyOeI0IaemOrayKaeiikaGIaemiEaG3aaSbaaSqaaiabdQgaQbqabaGccqGGPaqkcqGHsislcqWGubavcqGGOaakcqWG4baEdaWgaaWcbaGaemyAaKgabeaakiabgkHiTiabdIha4naaBaaaleaacqWGQbGAaeqaaOGaeiykaKIaeiykaKcabaaabaaabaGaey4kaSYaaSaaaeaacqWFbpGCaeaacqWFZoWzaaGaeiikaGIaemiEaG3aaSbaaSqaaiabdMgaPbqabaGccqGGOaakcqWG0baDcqGGPaqkcqGHsislcqWG4baEdaWgaaWcbaGaemOAaOgabeaakiabcIcaOiabdsha0jabcMcaPiabcMcaPmaaCaaaleqabaGaemivaqfaaOGaeiikaGIaemiEaG3aaSbaaSqaaiabdMgaPbqabaGccqGGOaakcqWG0baDcqGGPaqkcqGHsislcqWG4baEdaWgaaWcbaGaemOAaOgabeaakiabcIcaOiabdsha0jabcMcaPiabcMcaPiabc2faDbqaaaqaaaqaaiabgUcaRiabikdaYiabdIha4naaCaaaleqabaGaemivaqfaaOGaeiikaGIaemiDaqNaeiykaKIaeiikaGIaemyvauLaey4LIqSaemiuaaLaeiykaKIaeiikaGIaem4raCKaey4LIqSaemiraqKaey4kaSIaemysaKKaey4LIqSaemivaqLaeiykaKIaemiEaGNaeiikaGIaemiDaqNaeiykaKIaeiyFa0NaeiOla4caaaaa@8141@

If **E**(*S*_1_) < 0, we will have *J*(*t*) < 0, and thus, (2) follows immediately from (3).

### D. Proof of proposition 1

Proposition 1 can be proved by replacing *F *in *S*_2 _of (3) by the dynamics of (5), and using the sector conditions (6). We have

S2≡2(y1(t)−y2(t))TP[(A−T)(y1(t)−y2(t))+B1(f(y1(t))−f(y2(t)))−B2(g(y1(t))−g(y2(t)))]+ργ(y1(t)−y2(t))T(y1(t)−y2(t))≤2(y1(t)−y2(t))TP(A−T)(y1(t)−y2(t))+ργ(y1(t)−y2(t))T(y1(t)−y2(t))+2(y1(t)−y2(t))TPB1(f(y1(t))−f(y2(t)))−2(y1(t)−y2(t))TPB2(g(y1(t))−g(y2(t)))−2∑l=1nλ1l(fl(y1l(t))−fl(y2l(t)))⋅[(fl(y1l(t))−fl(y2l(t)))−k1(y1l(t)−y2l(t))]−2∑l=1nλ2l(gl(y1l(t))−gl(y2l(t)))⋅[(gl(y1l(t))−gl(y2l(t)))−k2(y1l(t)−y2l(t))].
 MathType@MTEF@5@5@+=feaafiart1ev1aaatCvAUfKttLearuWrP9MDH5MBPbIqV92AaeXatLxBI9gBaebbnrfifHhDYfgasaacH8akY=wiFfYdH8Gipec8Eeeu0xXdbba9frFj0=OqFfea0dXdd9vqai=hGuQ8kuc9pgc9s8qqaq=dirpe0xb9q8qiLsFr0=vr0=vr0dc8meaabaqaciaacaGaaeqabaqabeGadaaakeaafaqadeWcdaaaaaqaaiabdofatnaaBaaaleaacqaIYaGmaeqaaaGcbiqaaSWbcqGHHjIUaeaacqaIYaGmcqGGOaakcqWG5bqEdaWgaaWcbaGaeGymaedabeaakiabcIcaOiabdsha0jabcMcaPiabgkHiTiabdMha5naaBaaaleaacqaIYaGmaeqaaOGaeiikaGIaemiDaqNaeiykaKIaeiykaKYaaWbaaSqabeaacqWGubavaaGccqWGqbaucqGGBbWwcqGGOaakcqWGbbqqcqGHsislcqWGubavcqGGPaqkcqGGOaakcqWG5bqEdaWgaaWcbaGaeGymaedabeaakiabcIcaOiabdsha0jabcMcaPiabgkHiTiabdMha5naaBaaaleaacqaIYaGmaeqaaOGaeiikaGIaemiDaqNaeiykaKIaeiykaKcabaaabaaabaGaey4kaSIaemOqai0aaSbaaSqaaiabigdaXaqabaGccqGGOaakcqWGMbGzcqGGOaakcqWG5bqEdaWgaaWcbaGaeGymaedabeaakiabcIcaOiabdsha0jabcMcaPiabcMcaPiabgkHiTiabdAgaMjabcIcaOiabdMha5naaBaaaleaacqaIYaGmaeqaaOGaeiikaGIaemiDaqNaeiykaKIaeiykaKIaeiykaKIaeyOeI0IaemOqai0aaSbaaSqaaiabikdaYaqabaGccqGGOaakcqWGNbWzcqGGOaakcqWG5bqEdaWgaaWcbaGaeGymaedabeaakiabcIcaOiabdsha0jabcMcaPiabcMcaPiabgkHiTiabdEgaNjabcIcaOiabdMha5naaBaaaleaacqaIYaGmaeqaaOGaeiikaGIaemiDaqNaeiykaKIaeiykaKIaeiykaKIaeiyxa0fabaaabaaabaGaey4kaSYaaSaaaeaaiiGacqWFbpGCaeaacqWFZoWzaaGaeiikaGIaemyEaK3aaSbaaSqaaiabigdaXaqabaGccqGGOaakcqWG0baDcqGGPaqkcqGHsislcqWG5bqEdaWgaaWcbaGaeGOmaidabeaakiabcIcaOiabdsha0jabcMcaPiabcMcaPmaaCaaaleqabaGaemivaqfaaOGaeiikaGIaemyEaK3aaSbaaSqaaiabigdaXaqabaGccqGGOaakcqWG0baDcqGGPaqkcqGHsislcqWG5bqEdaWgaaWcbaGaeGOmaidabeaakiabcIcaOiabdsha0jabcMcaPiabcMcaPaqaaaqaaiabgsMiJcqaaiabikdaYiabcIcaOiabdMha5naaBaaaleaacqaIXaqmaeqaaOGaeiikaGIaemiDaqNaeiykaKIaeyOeI0IaemyEaK3aaSbaaSqaaiabikdaYaqabaGccqGGOaakcqWG0baDcqGGPaqkcqGGPaqkdaahaaWcbeqaaiabdsfaubaakiabdcfaqjabcIcaOiabdgeabjabgkHiTiabdsfaujabcMcaPiabcIcaOiabdMha5naaBaaaleaacqaIXaqmaeqaaOGaeiikaGIaemiDaqNaeiykaKIaeyOeI0IaemyEaK3aaSbaaSqaaiabikdaYaqabaGccqGGOaakcqWG0baDcqGGPaqkcqGGPaqkaeaaaeaaaeaacqGHRaWkdaWcaaqaaiab=f8aYbqaaiab=n7aNbaacqGGOaakcqWG5bqEdaWgaaWcbaGaeGymaedabeaakiabcIcaOiabdsha0jabcMcaPiabgkHiTiabdMha5naaBaaaleaacqaIYaGmaeqaaOGaeiikaGIaemiDaqNaeiykaKIaeiykaKYaaWbaaSqabeaacqWGubavaaGccqGGOaakcqWG5bqEdaWgaaWcbaGaeGymaedabeaakiabcIcaOiabdsha0jabcMcaPiabgkHiTiabdMha5naaBaaaleaacqaIYaGmaeqaaOGaeiikaGIaemiDaqNaeiykaKIaeiykaKcabaaabaaabaGaey4kaSIaeGOmaiJaeiikaGIaemyEaK3aaSbaaSqaaiabigdaXaqabaGccqGGOaakcqWG0baDcqGGPaqkcqGHsislcqWG5bqEdaWgaaWcbaGaeGOmaidabeaakiabcIcaOiabdsha0jabcMcaPiabcMcaPmaaCaaaleqabaGaemivaqfaaOGaemiuaaLaemOqai0aaSbaaSqaaiabigdaXaqabaGccqGGOaakcqWGMbGzcqGGOaakcqWG5bqEdaWgaaWcbaGaeGymaedabeaakiabcIcaOiabdsha0jabcMcaPiabcMcaPiabgkHiTiabdAgaMjabcIcaOiabdMha5naaBaaaleaacqaIYaGmaeqaaOGaeiikaGIaemiDaqNaeiykaKIaeiykaKIaeiykaKcabaaabaaabaGaeyOeI0IaeGOmaiJaeiikaGIaemyEaK3aaSbaaSqaaiabigdaXaqabaGccqGGOaakcqWG0baDcqGGPaqkcqGHsislcqWG5bqEdaWgaaWcbaGaeGOmaidabeaakiabcIcaOiabdsha0jabcMcaPiabcMcaPmaaCaaaleqabaGaemivaqfaaOGaemiuaaLaemOqai0aaSbaaSqaaiabikdaYaqabaGccqGGOaakcqWGNbWzcqGGOaakcqWG5bqEdaWgaaWcbaGaeGymaedabeaakiabcIcaOiabdsha0jabcMcaPiabcMcaPiabgkHiTiabdEgaNjabcIcaOiabdMha5naaBaaaleaacqaIYaGmaeqaaOGaeiikaGIaemiDaqNaeiykaKIaeiykaKIaeiykaKcabaaabaaabaGaeyOeI0IaeGOmaiZaaabmaeaacqWF7oaBdaWgaaWcbaGaeGymaeJaemiBaWgabeaaaeaacqWGSbaBcqGH9aqpcqaIXaqmaeaacqWGUbGBa0GaeyyeIuoakiabcIcaOiabdAgaMnaaBaaaleaacqWGSbaBaeqaaOGaeiikaGIaemyEaK3aaSbaaSqaaiabigdaXiabdYgaSbqabaGccqGGOaakcqWG0baDcqGGPaqkcqGGPaqkcqGHsislcqWGMbGzdaWgaaWcbaGaemiBaWgabeaakiabcIcaOiabdMha5naaBaaaleaacqaIYaGmcqWGSbaBaeqaaOGaeiikaGIaemiDaqNaeiykaKIaeiykaKIaeiykaKcabaaabaaabaGaeyyXICTaei4waSLaeiikaGIaemOzay2aaSbaaSqaaiabdYgaSbqabaGccqGGOaakcqWG5bqEdaWgaaWcbaGaeGymaeJaemiBaWgabeaakiabcIcaOiabdsha0jabcMcaPiabcMcaPiabgkHiTiabdAgaMnaaBaaaleaacqWGSbaBaeqaaOGaeiikaGIaemyEaK3aaSbaaSqaaiabikdaYiabdYgaSbqabaGccqGGOaakcqWG0baDcqGGPaqkcqGGPaqkcqGGPaqkcqGHsislcqWGRbWAdaWgaaWcbaGaeGymaedabeaakiabcIcaOiabdMha5naaBaaaleaacqaIXaqmcqWGSbaBaeqaaOGaeiikaGIaemiDaqNaeiykaKIaeyOeI0IaemyEaK3aaSbaaSqaaiabikdaYiabdYgaSbqabaGccqGGOaakcqWG0baDcqGGPaqkcqGGPaqkcqGGDbqxaeaaaeaaaeaacqGHsislcqaIYaGmdaaeWaqaaiab=T7aSnaaBaaaleaacqaIYaGmcqWGSbaBaeqaaOGaeiikaGIaem4zaC2aaSbaaSqaaiabdYgaSbqabaGccqGGOaakcqWG5bqEdaWgaaWcbaGaeGymaeJaemiBaWgabeaakiabcIcaOiabdsha0jabcMcaPiabcMcaPiabgkHiTiabdEgaNnaaBaaaleaacqWGSbaBaeqaaOGaeiikaGIaemyEaK3aaSbaaSqaaiabikdaYiabdYgaSbqabaGccqGGOaakcqWG0baDcqGGPaqkcqGGPaqkcqGGPaqkaSqaaiabdYgaSjabg2da9iabigdaXaqaaiabd6gaUbqdcqGHris5aaGcbaaabaaabaGaeyyXICTaei4waSLaeiikaGIaem4zaC2aaSbaaSqaaiabdYgaSbqabaGccqGGOaakcqWG5bqEdaWgaaWcbaGaeGymaeJaemiBaWgabeaakiabcIcaOiabdsha0jabcMcaPiabcMcaPiabgkHiTiabdEgaNnaaBaaaleaacqWGSbaBaeqaaOGaeiikaGIaemyEaK3aaSbaaSqaaiabikdaYiabdYgaSbqabaGccqGGOaakcqWG0baDcqGGPaqkcqGGPaqkcqGGPaqkcqGHsislcqWGRbWAdaWgaaWcbaGaeGOmaidabeaakiabcIcaOiabdMha5naaBaaaleaacqaIXaqmcqWGSbaBaeqaaOGaeiikaGIaemiDaqNaeiykaKIaeyOeI0IaemyEaK3aaSbaaSqaaiabikdaYiabdYgaSbqabaGccqGGOaakcqWG0baDcqGGPaqkcqGGPaqkcqGGDbqxcqGGUaGlaaaaaa@F8F6@

By letting *Q *= *PT *and denoting the first matrix in (8) by *M*_1_, we have *S*_2 _≤ *ξ*(*t*)*M*_1_*ξ*(*t*) < 0 for all *y*_1_, *y*_2 _except for *y*_1 _= *y*_2_, where *ξ*(*t*) = [(*y*_1_(*t*) - *y*_2_(*t*))^*T*^, (*f*(*y*_1_(*t*) - *f*(*y*_2_(*t*)))^*T*^, (*g*(*y*_1_(*t*) - *g*(*y*_2_(*t*)))^*T*^]^*T *^∈ *R*^3*n *× 1^. So, the first condition in (3) is satisfied. Substituting *Q *= *PT*, the second inequality in (3) is equivalent to the second inequality in (8). Thus, Proposition 1 is proved.

## Authors' contributions

CL gave the topic, developed theoretical results, designed the numerical experiments, analyzed the data, contributed materials/analysis tools. CL, LC and KA wrote the paper.
